# Face-to-Face Versus Digital, Telephone-Delivered, and Self-Help Cognitive Behavioral Therapy for Irritable Bowel Syndrome: Systematic Review and Bayesian Indirect Treatment Comparison Meta-Analysis

**DOI:** 10.2196/75833

**Published:** 2026-01-08

**Authors:** Qing-Feng Tao, Can Hua, Xiao Zhuo, Jian-Jiao Mou, Chao-Rong Xie, Yu-Xin Zhang, Bei Lv, Xin-Ying Niu, Min Chen, Hui Zheng

**Affiliations:** 1 Acupuncture and Tuina School, Chengdu University of Traditional Chinese Medicine, No.1166 Liutai Avenue, Wenjiang District, Chengdu, 611100, China, +86 28-61800000; 2 Department of Traditional Chinese Medicine, Dazhou Dachuan District People's Hospital (Dazhou Third People's Hospital), Dazhou, China; 3 Department of Colorectal Diseases, Hospital of Chengdu University of Traditional Chinese Medicine, Chengdu, China

**Keywords:** cognitive behavioral therapy, digital CBT, irritable bowel syndrome, indirect treatment comparison, meta-analysis

## Abstract

**Background:**

Cognitive behavioral therapy (CBT) is recommended for irritable bowel syndrome (IBS). However, it remains unclear whether face-to-face CBT is as effective as digital, self-help, or telephone-delivered CBT for IBS.

**Objective:**

This study aimed to estimate the relative effects of face-to-face CBT compared with digital, telephone-delivered, and self-help CBT for IBS and to assess whether there are adequate effective sample sizes to support the findings.

**Methods:**

Ovid MEDLINE, Embase, and the Cochrane Library were searched up to September 27, 2025. Randomized controlled trials of face-to-face, digital, self-help, or telephone-delivered CBT for IBS in adults were included. The primary outcome was the IBS symptom severity scale. The secondary outcomes were IBS quality of life and abdominal pain intensity. A Bayesian random effects model was used for the meta-analysis. The effective and required sample sizes were calculated to estimate whether the sample sizes were adequate. The certainty of evidence was evaluated using the Confidence in Network Meta-Analysis Framework. The risk of bias of included studies was assessed using the Cochrane Collaboration’s risk of bias tool (version 2).

**Results:**

We analyzed 22 studies involving 3161 participants. The number of participants ranged between 28 and 558. The mean (SD) age of participants was 37.2 (10.6) years, and 78.6% (2485/3161) were women. These randomized controlled trials were published between 2003 and 2025. We found that face-to-face CBT had similar effects compared with digital CBT (mean difference [MD] −0.89, 95% credible interval [CrI] −20.78 to 18.73), self-help CBT (MD −1.73, 95% CrI −21.03 to 17.80), and telephone-delivered CBT (MD −0.76, 95% CrI −20.86 to 19.38) in improving IBS symptom severity scale scores. The comparison between face-to-face CBT and self-help CBT had sufficient effective sample sizes (375/140), whereas the effective sample sizes for comparisons with digital CBT (347/729) and telephone-delivered CBT (140/627) were insufficient. The certainty of evidence was moderate to low. Similarly, in improving quality of life and abdominal pain intensity, face-to-face CBT showed equal effect compared with digital and self-help CBT, with insufficient sample sizes and low to very low evidence certainty.

**Conclusions:**

This is the first Bayesian meta-analysis to incorporate effective and required sample size calculations for comparisons among CBT modalities in IBS. We analyzed continuous data of the outcomes. Meanwhile, we computed the effective and required sample sizes, thereby quantifying the informational adequacy of each comparison. Our Bayesian meta-analysis demonstrated significant potential for digital, self-help, and telephone-delivered CBT for patients with IBS, but the effective sample sizes of most comparisons were inadequate. Digital, self-help, and telephone-delivered CBT can serve as important options for managing IBS in clinical practice. Given high heterogeneity, high risk of bias, and inadequate effective sample sizes, more high-quality studies are warranted.

## Introduction

### Rationale

Irritable bowel syndrome (IBS) is a prevalent disorder of brain-gut interaction marked by recurrent abdominal pain and altered bowel habits [1]. It affects 5% to 10% of the global population [2], significantly reducing quality of life, productivity, and mental health [3-6]. IBS causes a huge burden with years lived with disability at 627 per 100,000 [7]. The condition imposes substantial economic burdens, with direct costs approximately US $1 billion and indirect costs reaching US $50 million [8].

Research has shown the effectiveness of brain-gut behavioral therapies in improving IBS symptoms and quality of life [9-11]. Cognitive behavioral therapy (CBT), which integrates cognitive and behavioral techniques, is one such approach for alleviating symptoms [12]. Previous randomized controlled trials (RCTs) and meta-analyses have demonstrated that face-to-face CBT effectively relieves gastrointestinal symptoms and enhances quality of life [13-15]. However, despite the robust evidence supporting face-to-face CBT, its implementation is limited by the need for skilled mental health providers and the time and financial burden it imposes on patients [16,17]. In regions with limited CBT availability, accessing face-to-face sessions can be challenging. Consequently, there is a need for effective, accessible, and cost-effective alternative treatments.

The advent of the internet and digital technologies has provided a promising solution to the challenges associated with traditional face-to-face CBT for IBS. Digital CBT, which incorporates interactive programs based on cognitive behavioral models specific to IBS, has been increasingly integrated into medical practice [18,19]. Additionally, telephone-delivered CBT and self-help CBT have emerged as alternative delivery methods for IBS treatment. While RCTs have explored the clinical efficacy of these digital and remote CBT approaches, most have compared them to usual care or waiting lists [19,20], with only a few studies directly comparing them to face-to-face CBT [18,21,22]. As a result, the relative effectiveness of face-to-face CBT versus digital, telephone-delivered, and self-help CBT in managing IBS remains unclear.

Black et al [23] have conducted a network meta-analysis to evaluate the efficacy of psychological therapies for IBS. They found that face-to-face, digital, telephone-delivered, and self-help CBT were all efficacious for global IBS symptoms or abdominal pain, but none was superior to the others. Similarly, Goodoory et al [15] have explored the brain-gut behavioral treatments for abdominal pain in patients with IBS. Their results also found that face-to-face, digital, telephone-delivered, and self-help CBT were all effective for overall abdominal pain, with no one approach superior to another. However, both studies dichotomized outcomes—abdominal pain or global IBS symptoms were classified as *improved* or *not improved*—a strategy that can result in loss of information, reduced statistical power, and an increased risk of false-positive findings [24]. Therefore, the relative effectiveness of these distinct CBT modalities for IBS management requires further validation.

Moreover, studies using active controls require a large number of participants to achieve sufficient statistical power, counteracting inherent expectation biases and biases introduced by blinding [16]. Previous meta-analyses reported comparative effect sizes without evaluating whether the sample sizes were sufficient to yield stable estimates.

Indirect treatment comparison (ITC) allows us to evaluate the relative effectiveness of different interventions by fully using existing studies when there is no or insufficient direct evidence [25]. In addition, calculating the effective sample sizes and required sample sizes for each comparison can further validate the robustness of the findings [26].

### Objectives

Consequently, we conducted a Bayesian ITC meta-analysis with two primary objectives: (1) to estimate the relative effect of face-to-face CBT versus digital, telephone-delivered, and self-help CBT for IBS; and (2) to assess whether there are adequate effective sample sizes to support the findings.

## Methods

### Ethical Considerations

Our study was a systematic review and ITC meta-analysis of published studies and was exempt from review and approval by the research ethics committee. Ethical approval and consent to participate were acquired by each included study.

### Protocol and Registration

The systematic review was designed, conducted, and reported following the PRISMA-NMA (Preferred Reporting Items for Systematic Reviews and Meta-Analyses for Network Meta-Analyses) guidelines [27] and the PRISMA-S (Preferred Reporting Items for Systematic reviews and Meta-Analyses literature search extension) guidelines (Checklist 1) [28]. The systematic review had been previously registered on the Open Science Framework [29].

### Eligibility Criteria

The inclusion criteria were as follows. (1) participants—adults (aged ≥18 y) diagnosed with IBS according to the Rome or Manning criteria; (2) interventions—at least one CBT intervention, categorized into four distinct delivery modalities by referring to a previous study [30]: (i) face-to-face CBT (therapist-guided, in-person sessions conducted individually or in groups), (ii) digital CBT (therapist guided, provided via web-based platforms or mobile apps), (iii) telephone-delivered CBT (therapist guided, administered via telephone), and (iv) self-help CBT (therapist guided or unguided, structured written or web-based self-help materials that patients use to implement CBT techniques independently; the definitions of different CBT interventions are presented in Table S4 in Multimedia Appendix 1); (3) comparisons—placebo, waiting list, usual care, or other active treatments; (4) outcomes—at least one of the following outcomes: IBS symptom severity scale (IBS-SSS), IBS quality of life (IBS-QOL), and abdominal pain intensity (API); and (4) study design—a parallel-design RCT or a crossover-design RCT with available first-period data.

The exclusion criteria were as follows: (1) full-text articles unavailable and (2) duplicate articles.

### Information Sources and Search

MEDLINE, Embase, and the Cochrane Library were systematically searched from inception to September 29, 2024. A supplementary search was conducted on September 27, 2025. No single platform was used to search multiple databases. Medline was accessed via Ovid, whereas Embase and the Cochrane Library were searched through their respective official websites. There was no language restriction. Search terms encompassed *cognitive behavioral therapy* and *irritable bowel syndrome*. The search strategies are detailed in Table S1‐S3 in Multimedia Appendix 1. Our search strategies were not informed by previous reviews and were not peer-reviewed. Simultaneously, clinicaltrials.gov was also searched for potentially eligible studies. In addition, the reference lists of previous meta-analyses were screened for any eligible studies [15,23]. We did not search other online resources, browse websites, contact authors or experts, reach out to manufacturers, or use any other methods to obtain additional literature.

### Study Selection

Search results were imported into Zotero (version 7.0.8; Corporation for Digital Scholarship). Duplicate records were removed manually. Then, two reviewers (QF-T and CH) independently scanned the title and abstract. Subsequently, they assessed the full texts of potential RCTs for eligibility. Any disagreements were resolved through discussion, with a third reviewer (HZ) consulted if necessary.

### Outcome Assessments

The primary outcome was the change in IBS-SSS score. The secondary outcomes were the changes in IBS-QOL score and API score (measured by a visual analog scale or a gastrointestinal symptom diary for abdominal pain). Outcomes were evaluated at the end of treatment.

### Data Collection Process and Data Items

A reviewer (QF-T) extracted data using a standardized form, and the data were checked by the second reviewer (CH) independently. The extracted items included characteristics of the included RCTs, specifics of the intervention and control groups, and outcomes data. Data extraction for the meta-analysis used within-group change scores and their corresponding SDs for continuous outcomes. Disagreements were addressed through discussion and were consulted with a third reviewer (HZ). For missing data, we attempted to obtain them by reviewing previous studies.

### Risk of Bias Assessment

Risk of bias was assessed by two reviewers (JJ-M and XZ) independently using the Cochrane Collaboration’s risk of bias tool (version 2) for randomized trials [31]. By evaluating the following five components of the problem, that is, randomization process, deviations from intended interventions, missing outcome data, measurement of the outcome, and selection of the reported result, each included RCT was assessed as having low risk, some concerns, or high risk of bias.

### Statistical Analysis

#### Geometry of the Network

A network plot was generated in which the nodes represent the interventions and the edges represent the direct comparisons from RCTs. The size of each node was proportional to the number of participants receiving the intervention, and the thickness of the edges reflected the number of studies contributing to each direct comparison.

#### Summary Measures

As all the outcomes were continuous, the pooled effect size of the Bayesian analysis was estimated as mean difference (MD) with 95% credible intervals (CrIs). The effect size is the pooled estimate from a network meta-analysis that combines both direct and indirect evidence for each intervention. In addition, the surface under the cumulative rank curve (SUCRA) values were also calculated to determine the relative ranking of each intervention, with higher SUCRA values signifying a more favorable ranking for the intervention.

#### Planned Methods of Analysis

This systematic review was performed under a Bayesian framework with vague priors, which facilitates the integration of existing evidence with new data to update estimates of treatment effects and provide superior handling of uncertainty in small sample studies compared to traditional approaches. We assessed the fit of the random effects (REs) and fixed effects (FEs) models by examining their posterior total residual deviance, the deviance information criterion (DIC), and the number of unconstrained data points. A model was considered to have a better fit if it exhibited a posterior total residual deviance closer to the number of unconstrained data points and a lower DIC.

When two or more arms received the same intervention, we pooled their participants, means, and SD. Meanwhile, we pooled mindfulness, stress management, and education as an alternative psychotherapy. On the basis of the actual delivery methods used in the study, we categorized these interventions separately as alternative face-to-face psychotherapy, alternative self-help psychotherapy, or alternative digital psychotherapy. τ^2^ was used to estimate the heterogeneity among RCTs, with a τ^2^ of more than 0.36 indicating significant heterogeneity [23].

#### Assessment of Inconsistency

An unrelated mean effects model was fit by drawing the dev-dev plots to assess the inconsistency globally, and node-splitting was fit to assess the inconsistency locally for each potentially inconsistent comparison in turn.

#### Additional Analyses

Two sensitivity analyses were conducted to calculate the robustness of the results: (1) excluding the RCT that was rated as high risk of bias and (2) using frequentist framework as the statistical method; To explore the source of heterogeneity, we performed subgroup analyses according to treatment duration (<8 wk or ≥8 wk), the delivery format of face-to-face CBT (individual vs group), and the guidance level of self-help CBT (guided vs unguided) using the primary outcome data.

All the analyses were conducted in R version 4.3.1. The Bayesian analysis was performed using the *multinma* package, and the frequentist analysis used the *netmeta* package.

### The Certainty of Evidence

The certainty of evidence of each outcome was graded using a web application—the Confidence in Network Meta-Analysis Framework [32]. This approach evaluated six domains, such as within-study bias, reporting bias, indirectness, imprecision, heterogeneity, and incoherence. The certainty of evidence of each outcome was graded as high, moderate, low, or very low.

### The Estimate of Effective Sample Sizes and Required Sample Sizes

The effective number of trials and the effective sample sizes for the ITC were calculated using the method developed by Thorlund and Mills [26]. The effective number of trials represents the number of trials required in an ITC to achieve a comparable level of power and precision to that of a single direct head-to-head trial. The effective sample sizes denote the number of participants in the comparison that would provide the same degree and strength of evidence as that provided in an RCT. Additionally, the required sample sizes were estimated [33]. In this systematic review, a noninferiority design with 1:1 randomization was used to estimate the required sample sizes. If the effective sample sizes reach the required sample sizes, it would demonstrate the robustness of the findings; otherwise, further research is needed to confirm the findings.

## Results

### Study Selection

**Figure 1.**
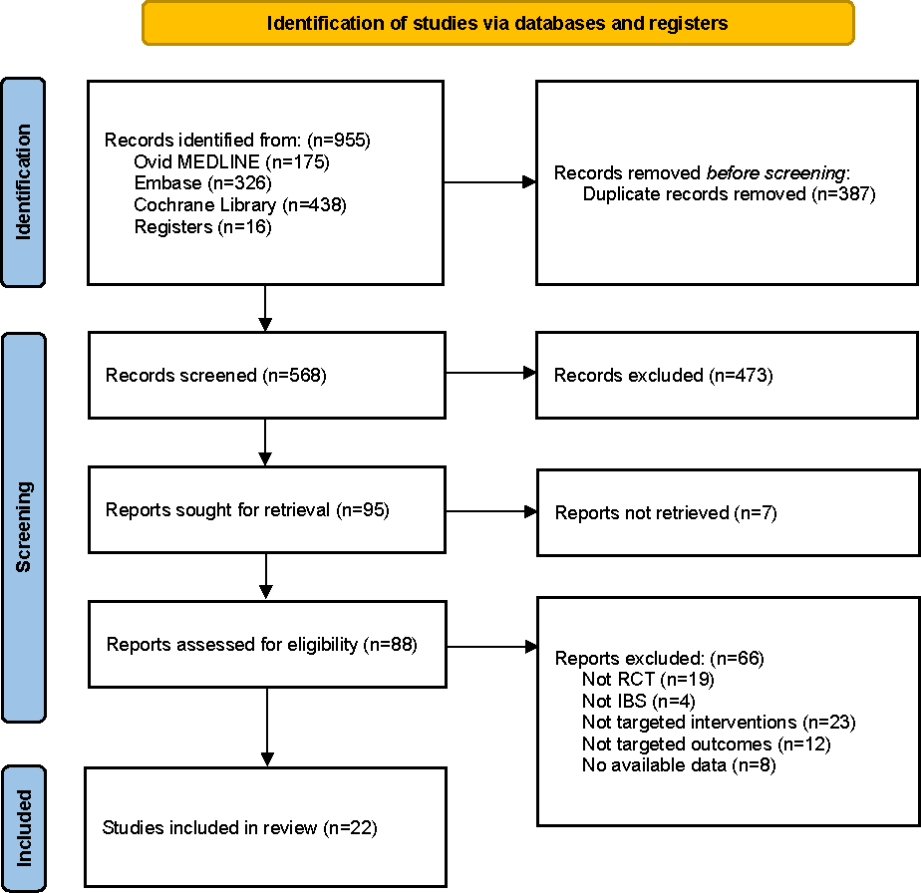
PRISMA (Preferred Reporting Items for Systematic Reviews and Meta-Analyses) flow diagram of the literature search and study selection. IBS: irritable bowel syndrome; RCT: randomized controlled trial.

The initial database and registry searches identified 955 articles (Figure 1). After screening the titles and abstracts of 568 articles, 473 were excluded. Following a full-text review of 88 articles, 22 eligible RCTs were included [13,14,18-22,34-48].

### Study Characteristics

The characteristics of the eligible RCTs are presented in Table 1. A total of 3161 participants were enrolled in these RCTs, and the number of participants ranged between 28 and 558. The mean (SD) age of participants was 37.2 (10.6) years, and 78.6% were women. The RCTs included in our study were published between 2003 and 2025. Among these RCTs, 8 were from the United States (36.4%), 5 (22.7%) were from Sweden, 2 (9.1%) each from the United Kingdom, Korea, and Iran, and 1 (4.5%) each from the Netherlands, Canada, and Japan. In terms of intervention types, 7 (31.8%) RCTs reported face-to-face CBT, 6 (27.3%) RCTs reported digital CBT, 4 (18.2%) RCTs reported self-help CBT, 2 (9.1%) RCTs assessed both digital CBT and self-help CBT, 2 (9.1%) RCTs evaluated both self-help CBT and face-to-face CBT, and another (4.5%) examined both digital CBT and telephone-delivered CBT. All studies adopted the Rome criteria as the diagnostic standard. Two RCTs specifically focused on diarrhea-predominant IBS, while the remaining studies included all IBS subtypes.

**Table 1.** Characteristics of the included studies.

Study, year	Country	Study design	Disease	Diagnostic criteria	Interventions	Treatment duration (wk)	Population [sample size (n), female (%), mean ages (y)]	Results
Dehkordi and Solati, 2017 [34]	Iran	RCT^a^	IBS-D^b^	Rome III	Face-to-face CBT^c^ versus usual care	8	64, 63, 33.7	IBS-QOL^d^: face-to-face CBT>usual care
Everitt et al, 2019 [35]	UK	RCT	IBS^e^	Rome III	Digital CBT versus telephone-delivered CBT versus usual care	48	558, 76, 43.1	IBS-SSS^f^: digital CBT=telephone-delivered CBT>usual care
Haghayegh et al, 2011 [36]	Iran	RCT	IBS-D	Rome II	Face-to-face CBT versus waiting list	8	32, 46, N/A^g^	IBS-QOL: face-to-face CBT>waiting list
Hunt et al, 2009 [38]	USA	RCT	IBS	Rome II	Digital CBT versus waiting list	6	54, 81, 38.5	IBS-QOL: digital CBT>waiting list
Hunt et al, 2015 [37]	USA	RCT	IBS	Rome III	Self-help CBT versus waiting list	6	60, 83, 36	IBS-QOL: self-help CBT>waiting list
Hunt et al, 2021 [20]	USA	RCT	IBS	Rome III	Self-help CBT versus waiting list	8	121, 75, 32	IBS-QOL: self-help CBT>waiting list
Hunt et al, 2025 [48]	USA	RCT	IBS	Rome IV	Self-help CBT versus alternative self-help psychotherapy	8	267, 72.3, 36.6	IBS-QOL: self-help CBT>alternative self-help psychotherapy
Jang et al, 2014 [39]	Korea	RCT	IBS	Rome III	Face-to-face CBT versus waiting list	8	90, 100, 21.6	IBS-QOL: face-to-face CBT>waiting list
Kennedy et al, 2005 [40]	UK	RCT	IBS	Rome I	Face-to-face CBT versus usual care	12	149, N/A, N/A	IBS-SSS: face-to-face CBT>usual care
Kikuchi et al, 2022 [13]	Japan	RCT	IBS	Rome III	Digital CBT versus face-to-face CBT versus usual care	10	114, 63, 39.7	IBS-SSS, IBS-QOL: digital CBT=face-to-face CBT>usual care
Lackner et al, 2007 [14]	USA	RCT	IBS	Rome II	Face-to-face CBT versus alternative face-to-face psychotherapy versus waiting list	10	147, 82, 49.9	IBS-QOL: face-to-face CBT=alternative face-to-face psychotherapy=waiting list
Lackner et al, 2008 [21]	USA	RCT	IBS	Rome II	Face-to-face CBT versus self-help CBT versus waiting list	10	75, 86, 46.6	IBS-SSS, IBS-QOL: face-to-face CBT=self-help CBT>waiting list
Lackner et al, 2018 [22]	USA	RCT	IBS	Rome III	Face-to-face CBT versus self-help CBT versus alternative face-to-face psychotherapy	10	436, 80, 41.4	IBS-SSS: Face-to-face CBT=self-help CBT=alternative face-to-face psychotherapy
Lindfors 2020 [19]	Sweden	RCT	IBS	Rome IV	Digital CBT versus face-to-face CBT	3	141, 80, 37	IBS-SSS, IBS-QOL, API^h^: Digital CBT=face-to-face CBT
Ljótsson et al, 2010 [44]	Sweden	RCT	IBS	Rome III	Digital CBT versus waiting list	10	86, 85, 34.6	IBS-QOL, API: Digital CBT>waiting list
Ljótsson et al, 2011a [42]	Sweden	RCT	IBS	Rome III	Digital CBT versus alternative digital psychotherapy	10	195, 79, 38.9	IBS-QOL, API: Digital CBT=alternative digital psychotherapy
Ljótsson et al, 2011b [[41]	Sweden	RCT	IBS	Rome III	Digital CBT versus waiting list	10	61, 74, 34.9	IBS-QOL: Digital CBT>waiting list
Ljótsson et al, 2014 [43]	Sweden	RCT	IBS	Rome III	Digital CBT versus alternative digital psychotherapy	10	311, 80, 42.4	IBS-QOL: Digital CBT=alternative digital psychotherapy
Oerlemans et al, 2011 [19]	Netherlands	RCT	IBS	Rome III	Digital CBT versus usual care	4	76, 84, 38.3	IBS-QOL, API: Digital CBT>usual care
Owusu et al, 2021 [45]	USA	RCT	IBS	Rome IV	Self-help CBT versus waiting list	12	36, 78, 39.2	IBS-SSS: self-help CBT>waiting list
Tkachuk et al, 2003 [46]	Canada	RCT	IBS	Rome II	Face-to-face CBT versus waiting list	9	28, 96, 39.5	API: face-to-face CBT=waiting list
Yang et al, 2022 [47]	Korea	RCT	IBS	Rome III	Face-to-face CBT versus waiting list	4	60, 88, 20.5	IBS-SSS, IBS-QOL: face-to-face CBT>waiting list

a RCT: randomized controlled trial.

b IBS-D: diarrhea-predominant irritable bowel syndrome.

c CBT: cognitive behavioral therapy.

d IBS-QOL: irritable bowel syndrome quality of life.

e IBS: irritable bowel syndrome.

f IBS-SSS: irritable bowel syndrome symptom severity scale.

g N/A: not available.

h API: abdominal pain intensity.

### Risk of Bias

**Figure 2.**
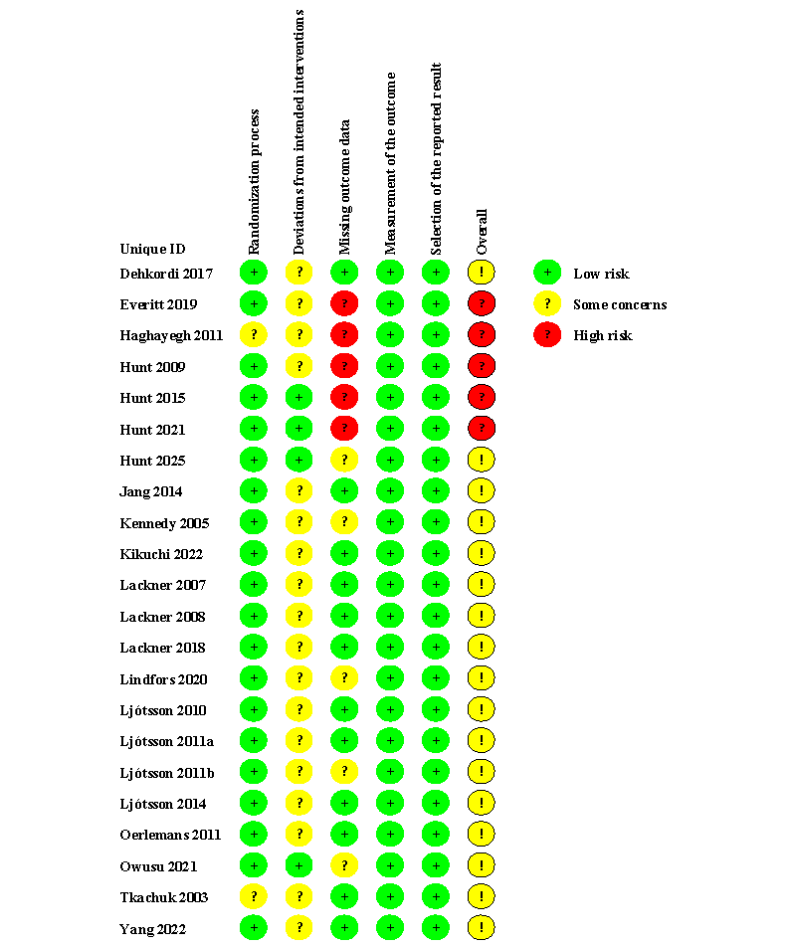
Risk of bias of included randomized controlled trials [13,14,18-22,34-48].

Risk of bias assessment is presented in Figure 2. Of the RCTs, 5 (22.7%) were adjudicated to be at high risk of bias, and 17 (77.3%) were adjudicated to have some concerns.

### Exploration for Model Fit and Inconsistency

The results of the total posterior residuals deviance, the number of data points, and the DIC for both RE and FE models across all outcomes are presented in Table S5 in Multimedia Appendix 1. The RE model demonstrated a residual deviance closer to the number of data points and exhibited a lower DIC. Moreover, dev-dev plots indicated that all points were approximately aligned with the equality line, suggesting no evidence of global inconsistency (Figure S1 in Multimedia Appendix 1). Furthermore, the results of the node-splitting analysis indicated no local inconsistency (Figure S2 in Multimedia Appendix 1). Consequently, RE consistency models were selected for conducting the analyses.

### Irritable Bowel Syndrome Symptom Severity Scale

**Figure 3.**
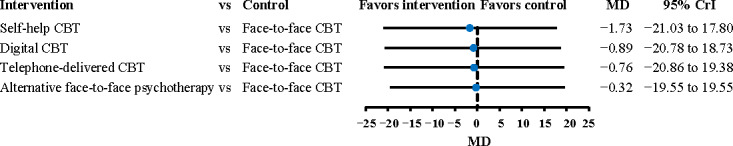
Effect of comparison between face-to-face CBT and other types of CBT for irritable bowel syndrome symptom severity scale [13,18,21,22,35,40,45,47]. The black vertical line corresponds to 0. CBT: cognitive behavioral therapy; CrI: credible interval; MD: mean difference.

A total of 8 RCTs, comprising 1562 participants, contributed data to the outcome analysis. The network plot is presented in Figure S3 in Multimedia Appendix 1. Bayesian model results indicated that face-to-face CBT had similar effects compared to digital, telephone-delivered, and self-help CBT in reducing IBS-SSS (self-help CBT: MD −1.73, 95% CrI −21.03 to 17.80; digital CBT: MD −0.89, 95% CrI −20.78 to 18.73; telephone-delivered CBT: MD −0.76, 95% CrI −20.86 to 19.38; τ²=24.61; Figure 3). The certainty of evidence was rated as moderate to low (Table S6 in Multimedia Appendix 1). The SUCRA rankings suggested that self-help CBT was the most effective (SUCRA 0.56, Table S7 in Multimedia Appendix 1). Furthermore, sensitivity analyses, which excluded high-risk RCTs (Figure S4 in Multimedia Appendix 1) and used a frequentist method (Figure S5 in Multimedia Appendix 1), yielded similar results, indicating the stability of the findings.

The results of the effective sample sizes and the required sample sizes are presented in Table 2 and Multimedia Appendix 2. Adequate effective sample sizes were observed for the comparison between face-to-face and self-help CBT (375/140, 267.9%; Table 2 and Multimedia Appendix 2). However, for the other comparisons, the effective sample sizes were insufficient, suggesting that more studies are needed in the future (digital CBT: 347/729, 47.6%; telephone-delivered CBT: 140/627, 22.3%; Table 2 and Multimedia Appendix 2).

**Table 2.** The effective sample sizes and required sample sizes estimation of outcomes.

Comparison (vs face-to-face CBT^a^)	Effective number of trials (n/N)	Effective head-to-head sample sizes, n	Effective indirect sample sizes, n	Total effective sample sizes, n	Required sample sizes, n	Information fraction (n/N,%)	Network meta-analysis, MD^b^ estimate (95% credible intervals)
IBS-SSS^c^							
Digital CBT	4/8	195	152	347	729	347/729, 47.6	−0.89 (−20.78 to 18.73)
Self-help CBT	4/8	339	36	375	140	375/140, 267.9	−1.73 (−21.03 to 17.80)
Telephone-delivered CBT	3/9	N/A^d^	140	140	627	140/627, 22.3	−0.76 (−20.86 to 19.38)
IBS-QOL^e^							
Digital CBT	8/9	195	107	302	3550	302/3550, 8.5	−4.29 (−19.10 to 10.59)
Self-help CBT	8/9	48	123	171	4610	171/4610, 3.7	−2.82 (−18.83 to 13.76)
API^f^							
Digital CBT	2/12	141	21	162	3764	162/3764, 4.3	−1.14 (−19.69 to 16.49)

a CBT: cognitive behavioral therapy.

b MD: mean difference.

c IBS-SSS: irritable bowel syndrome symptom severity scale.

d N/A: not applicable.

e IBS-QOL: irritable bowel syndrome quality of life.

f API: abdominal pain intensity.

### Irritable Bowel Syndrome Quality of Life

**Figure 4.**
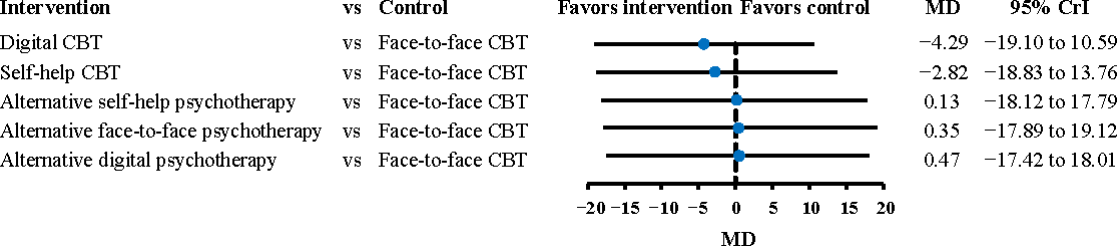
Effect of comparison between face-to-face CBT and other types of CBT for irritable bowel syndrome quality of life [13,14,18-21,34,36-39,41-44,47,48]. The black vertical line corresponds to 0. CBT: cognitive behavioral therapy; CrI: credible interval; MD: mean difference.

A total of 17 RCTs, involving 1798 participants, contributed data to the outcome analysis. The network plot is presented in Figure S6 in Multimedia Appendix 1. Face-to-face CBT was found to be equally effective as digital CBT and self-help CBT in enhancing the quality of life for patients with IBS (digital CBT: MD −4.29, 95% CrI −19.10 to 10.59; self-help CBT: MD −2.82, 95% CrI −18.83 to 13.76; τ²=15.93; Figure 4). The Confidence in Network Meta-Analysis Framework evidence rating was very low (Table S6 in Multimedia Appendix 1). Digital CBT ranked higher than face-to-face CBT, with a SUCRA value of 0.71 (Table S7 in Multimedia Appendix 1). Sensitivity analyses confirmed the stability of these results after excluding high-risk RCTs (Figure S7 in Multimedia Appendix 1) and using a frequentist method (Figure S8 in Multimedia Appendix 1).

The effective sample sizes did not meet the required sample sizes, suggesting that more studies are warranted (digital CBT: 302/3550, 8.5%; self-help CBT: 171/4610, 3.7%; Table 2 and Multimedia Appendix 2).

### Abdominal Pain Intensity

Four RCTs, involving 327 participants, contributed data to the outcome analysis. The network plot is presented in Figure S9 in Multimedia Appendix 1. Digital CBT showed similar effects compared with face-to-face CBT in reducing the API, with very low certainty of evidence (MD −1.14, 95% CrI −19.69 to 16.49, τ²=23.66; Figure S10 in Multimedia Appendix 1, Table S6 in Multimedia Appendix 1). The SUCRA rankings indicated that digital CBT was ranked higher than face-to-face CBT (SUCRA 0.54 vs 0.49, Table S7 in Multimedia Appendix 1). The result was consistent with the sensitivity analysis using a frequentist method (Figure S11 in Multimedia Appendix 1).

As presented in Table 2 and Multimedia Appendix 2, the effective sample sizes did not meet the required sample sizes in the comparison between face-to-face CBT and digital CBT (141/3764, 4.3%), suggesting that more studies are needed.

### Results of Subgroup Analysis

The results of the subgroup analysis of the primary outcome are presented in Figure S13‐S15 in Multimedia Appendix 1. We found that the duration of treatment (Figure S12 in Multimedia Appendix 1), the delivery method of face-to-face CBT (Figure S13 in Multimedia Appendix 1), or the guidance level of self-help CBT (Figure S14 in Multimedia Appendix 1) had no significant influence on the heterogeneity. Additionally, subgroup analysis suggested that the treatment duration (<8 wk or ≥8 wk; Figure S12 in Multimedia Appendix 1), the delivery method of face-to-face CBT (individual or group face-to-face CBT, Figure S13 in Multimedia Appendix 1), or the guidance level of self-help CBT (guided or unguided self-help CBT, Figure S14 in Multimedia Appendix 1) did not significantly modify the effect on global IBS symptoms.

## Discussion

### Summary of Evidence

In this systematic review, we assessed the relative effect of different delivery methods of CBT for patients with IBS and assessed the effective sample sizes and required sample sizes of each finding to evaluate the robustness. We included 22 RCTs involving 3161 adults with IBS. We found that face-to-face CBT presented a similar effect compared with self-help CBT in improving global IBS symptoms, with sufficient effective sample sizes. Face-to-face CBT showed an equal effect compared with digital CBT and telephone-delivered CBT in improving global IBS symptoms; however, the effective sample sizes were less than the required sample sizes. For quality of life and API, there were similar effects between face-to-face CBT and digital and self-help CBT, but the effective sample sizes were insufficient.

Face-to-face CBT is the primary mode of CBT delivery and is recognized as an effective intervention for various conditions, including major depressive disorder, insomnia, and IBS [1,49,50]. Our results showed that compared with face-to-face CBT, there were slight differences between digital, telephone-delivered, and self-help CBT in IBS-SSS, yet none reached the minimal clinically important difference value of 50 [51]. The similar effects might be explained by the fact that digital, telephone-delivered, and self-help CBT represent modifications of traditional face-to-face CBT in terms of delivery method. However, the core principles of CBT remain consistent across these modalities, ensuring efficacy while enhancing convenience and accessibility. Our findings are consistent with those of previous meta-analyses by Black et al [23] and Goodoory et al [15].

Compared to previous meta-analyses [15,23], their studies primarily used binary outcome measures and exclusively used frequentist analysis methods. In contrast, our study analyzed continuous outcomes for the IBS-SSS, IBS-QOL, and API. We used a Bayesian model for the primary analysis and a frequentist model for sensitivity analysis to confirm the stability of our findings. Additionally, our study is the first to incorporate effective and required sample size calculations for ITC within CBT for IBS. We conducted a novel assessment of effective and required sample sizes, which has not been previously undertaken in CBT studies for IBS. Our results indicate that there are sufficient effective sample sizes to support that there is a similar effect between face-to-face CBT and self-help CBT in improving global IBS symptoms. However, other comparisons exhibited insufficient effective sample sizes.

### Implication for Practice and Research

For clinical practice, the social and economic impact of IBS necessitates the demonstration of both clinical effectiveness and innovative direct-to-patient delivery systems to enhance patient access to appropriate therapeutic interventions [22]. Our findings indicate that digital, self-help, and telephone-delivered CBT have similar effects compared with face-to-face CBT. Meanwhile, a previous study has shown that a 10-week digital CBT treatment can reduce direct medical costs by US $358 and indirect costs by US $5014 [52]. Therefore, these alternative delivery methods deserve consideration in the clinical management of IBS. Notably, digital CBT has considerable potential for managing IBS because of its effectiveness [18], accessibility [38], and cost-effectiveness [52,53]. Additionally, our research found that both individualized face-to-face CBT and group face-to-face CBT demonstrated a similar effect in relieving the overall symptoms of patients with IBS compared to other treatments. Therefore, clinicians may choose between individual CBT and group CBT based on resource availability and patient preference in clinical practice.

Furthermore, for research, although our analysis suggests comparable effectiveness between digital CBT and face-to-face CBT, the effective sample size (n=347) represents only 47.6% of the required sample size (n=729) for this comparison. This indicates that while the current evidence is promising, implementation of digital CBT as a substitute for traditional face-to-face delivery in clinical practice would necessitate monitoring of larger patient cohorts to definitively confirm therapeutic equivalence and ensure consistent clinical outcomes.

### Limitations

Several limitations should be considered when interpreting our findings. At the study and outcome level, first, none of the included studies were rated as low risk of bias, primarily due to the difficulty of blinding in RCTs of CBT. Moreover, five studies (22.7%) were rated as high risk of bias; hence, we excluded RCTs with high risk of bias for sensitivity analysis and found that the results were consistent with the main analysis. Second, there was significant heterogeneity among the included studies; therefore, we conducted subgroup analyses on treatment duration, the delivery method of face-to-face CBT, and the guidance level of self-help CBT. These analyses revealed that these factors did not significantly influence the heterogeneity. Initially, we intended to explore whether the source of heterogeneity was related to disease duration, disease subtype, and gender through subgroup analyses. However, this analysis could not be conducted due to the limited number of included studies, which is also attributed to the scarcity of high-quality RCTs on CBT for IBS, further emphasizing the need for more high-quality RCTs.

At the review level, first, we searched only Ovid MEDLINE, Embase, Cochrane Library, and ClinicalTrials.gov. We did not pursue additional literature via other online resources, correspondence with authors or experts, contact with manufacturers, or any other means; thus, some literature may have been missed. Nevertheless, we screened the reference lists of previous meta-analyses for any missed studies. Second, our search strategies did not consult an information scientist, which is another limitation of our study. Third, when estimating effective sample sizes, we used an estimation method that was not adjusted for heterogeneity, which may have overestimated the effective sample sizes [26]. Nonetheless, our results indicated that even if the effective sample sizes were overestimated, they still fell short of the required sample sizes for most comparisons. This finding further underscores the necessity for additional RCTs to validate our results in the future.

### Conclusions

To our knowledge, this is the first Bayesian meta-analysis to incorporate effective and required sample size calculations for indirect treatment comparisons among CBT modalities in IBS. Compared with previous meta-analyses, we analyzed continuous outcomes of global symptoms, IBS-QOL, and API. For each comparison, we computed the effective sample size and the required sample size, thereby quantifying the informational adequacy. The meta-analysis suggested that digital, self-help, and telephone-delivered CBT had similar effects on global IBS symptoms, quality of life, and API as face-to-face CBT in patients with IBS. Therefore, digital CBT, self-help CBT, and telephone-delivered CBT can serve as important methods for managing IBS in clinical practice. However, our findings suggested that the effective sample sizes of most comparisons were insufficient. Given high heterogeneity, high risk of bias, and inadequate effective sample sizes, more high-quality studies are warranted in the future.

## Supplementary material

10.2196/75833 Multimedia Appendix 1 Search strategies, definition of different cognitive behavioral therapy, model fit, certainty of evidence, consistency assessment, value of surface under the cumulative rank curve, network diagram, sensitivity analysis, and subgroup analysis.

10.2196/75833 Multimedia Appendix 2 Assessment of effective number of trials, effective sample sizes, and required sample sizes.

10.2196/75833 Checklist 1 PRISMA checklist.

## Abbreviations

API: abdominal pain intensity
CBT: cognitive behavioral therapy
CrI: credible interval
DIC: Deviance information criteria
FE: fixed effect
IBS: irritable bowel syndrome
IBS-QOL: irritable bowel syndrome quality of life
IBS-SSS: irritable bowel syndrome symptom severity scale
ITC: indirect treatment comparison
MD: mean difference
PRISMA-NMA: Preferred Reporting Items for Systematic Reviews and Meta-Analyses for Network Meta-Analyses
PRISMA-S: Preferred Reporting Items for Systematic reviews and Meta-Analyses literature search extension
RCT: randomized controlled trial
RE: random effect
SUCRA: Surface under the cumulative rank curve
