# Supplementary material for: Face-to-Face Versus Digital, Telephone-Delivered, and Self-Help Cognitive Behavioral Therapy for Irritable Bowel Syndrome: Systematic Review and Bayesian Indirect Treatment Comparison Meta-Analysis
Source: J Med Internet Res. 2026 Jan 8;28:e75833. doi: 10.2196/75833 (PMC12782461; doi:10.2196/75833)
Supplement: Multimedia Appendix 1 [file jmir-v28-e75833-s001.docx]

**Supplementary materials files**

Table S1. Search strategy in Ovid Medline 2

Table S2. Search strategy in Embase 3

Table S3. Search strategy in Cochrane Library 4

Table S4. Definition of different CBT 5

Table S5. Summary of fixed-effects and random-effects model fit statistics from network meta-analysis 6

Table S6. Certainty of evidence of outcomes 7

Table S7. Value of SUCRA for each treatment on outcomes 9

Figure S1. The “dev-dev” plots of random-effects consistency and inconsistency models for NMA by outcomes 10

Figure S2. The node-splitting plots of the posterior distributions of the direct, indirect, and network estimation 11

Figure S3. Network diagram of comparison of IBS-SSS 12

Figure S4. Sensitivity analysis of IBS-SSS after excluding RCTs at high risk of bias 13

Figure S5. Sensitivity analysis of IBS-SSS using frequentist methods 14

Figure S6. Network diagram of comparison of IBS-QOL 15

Figure S7. Sensitivity analysis of IBS-QOL after excluding RCTs at high risk of bias 16

Figure S8. Sensitivity analysis of IBS-QOL using frequentist methods 17

Figure S9. Network diagram of comparison of API 18

Figure S10. Effect of comparison between face-to-face CBT and digital CBT of API 19

Figure S11. Sensitivity analysis of API using frequentist methods 20

Figure S12. Subgroup analysis of IBS-SSS on treatment duration 21

Figure S13. Subgroup analysis of IBS-SSS on delivery method of face-to-face CBT 22

Figure S14. Subgroup analysis of IBS-SSS on the guidance level of self-help CBT 23

**Table S1.** Search strategy in Ovid Medline

| **ID** | **Search strategy** |
| --- | --- |
| 1 | randomized controlled trial.pt. |
| 2 | controlled clinical trial.pt. |
| 3 | randomized.ab,ti. |
| 4 | randomised.ab,ti. |
| 5 | randomly.ab,ti. |
| 6 | trial.ab,ti. |
| 7 | groups.ab,ti. |
| 8 | 1 or 2 or 3 or 4 or 5 or 6 or 7 |
| 9 | limit 8 to humans |
| 10 | exp irritable bowel syndrome/ |
| 11 | (irritable bowel syndrome or IBS).ab,ti. |
| 12 | 11 or 12 |
| 13 | exp cognitive behavioral therapy/ |
| 14 | (cognitive behavioural therapy or cognitive behavioral therapy or CBT or behavioural therapy or behavioral therapy).ab,ti. |
| 15 | 13 or 14 |
| 16 | 9 and 12 and 15 |

**Table S2.** Search strategy in Embase

| **ID** | **Search strategy** |
| --- | --- |
| 1 | 'randomized controlled trial'/exp |
| 2 | 'controlled clinical trial'/exp |
| 3 | randomized:ab,ti |
| 4 | randomised:ab,ti |
| 5 | randomly:ab,ti |
| 6 | trial:ab,ti |
| 7 | groups:ab,ti |
| 8 | #1 OR #2 OR #3 OR #4 OR #5 OR #6 OR #7 |
| 9 | (#1 OR #2 OR #3 OR #4 OR #5 OR #6 OR #7) AND [humans]/lim |
| 10 | 'irritable bowel syndrome'/exp |
| 11 | 'irritable bowel syndrome':ab,ti OR IBS:ab,ti |
| 12 | #10 OR #11 |
| 13 | 'cognitive behavioural therapy'/exp |
| 14 | 'cognitive behavioral therapy'/exp |
| 15 | CBT/exp |
| 16 | 'cognitive behavioural therapy':ab,ti OR 'cognitive behavioral therapy':ab,ti OR CBT:ab,ti OR 'behavioural therapy':ab,ti OR 'behavioral therapy':ab,ti |
| 17 | #13 OR #14 OR #15 OR #16 |
| 18 | #9 AND #12 AND #17 |

**Table S3.** Search strategy in Cochrane Library

| **ID** | **Search strategy** |
| --- | --- |
| 1 | (randomized controlled trial):pt |
| 2 | (controlled clinical trial):pt |
| 3 | (randomized):ti,ab,kw |
| 4 | (randomised):ti,ab,kw |
| 5 | (randomly):ti,ab,kw |
| 6 | (trial):ti,ab,kw |
| 7 | (groups):ti,ab,kw |
| 8 | #1 OR #2 OR #3 OR #4 OR #5 OR #6 OR #7 in Trials |
| 9 | MeSH descriptor: [irritable bowel syndrome] explode all trees |
| 10 | (irritable bowel syndrome OR IBS):ti,ab,kw |
| 11 | #9 OR #10 |
| 12 | MeSH descriptor: [cognitive behavioral therapy] explode all trees |
| 13 | (cognitive behavioural therapy OR cognitive behavioral therapy OR CBT OR behavioural therapy OR behavioural therapy):ti,ab,kw |
| 14 | #12 OR #13 |
| 15 | #8 AND #11 AND #14 |

**Table S4.** Definition of different CBT

| **Interventions** | **Definitions** |
| --- | --- |
| Face-to-face CBT | CBT is delivered face-to-face in clinics via scheduled, therapist-guided sessions conducted individually or in groups. |
| Digital CBT | CBT is delivered through web or mobile applications with therapist guidance. |
| Telephone-delivered CBT | CBT is delivered through telephone with therapist guidance. |
| Self-help CBT | CBT delivery relies mainly on structured written or web-based self-help materials, which may be therapist-guided or unguided. |

**Footnote:** CBT, cognitive behavioral therapy.

**Table S5.** Summary of fixed-effects and random-effects model fit statistics from network meta-analysis

| **Model** | **Number of data points** | **Posterior total residual deviance** | **DIC** |
| --- | --- | --- | --- |
| **IBS-SSS** | | | |
| **FE consistency** | 20 | 12.2 | 18.6 |
| **FE inconsistency** | 20 | 12.5 | 19.1 |
| **RE consistency** | 20 | 12.3 | 18.6 |
| **IBS-QOL** | | | |
| **FE consistency** | 35 | 20.2 | 39.7 |
| **FE inconsistency** | 35 | 22.1 | 41.1 |
| **RE consistency** | 35 | 20.8 | 40 |
| **API** | | | |
| **FE consistency** | 8 | 4.5 | 8.7 |
| **FE inconsistency** | 8 | 4.6 | 8.9 |
| **RE consistency** | 8 | 4.5 | 8.7 |

**Footnote:** DIC, deviance information criteria; IBS-SSS, irritable bowel syndrome symptom severity scale; FE, fixed-effect; RE, random-effect; IBS-QOL, irritable bowel syndrome quality of life; API, abdominal pain intensity.

**Table S6.** Certainty of evidence of outcomes

| Comparison | Number of studies | Within-study bias | Reporting bias | Indirectness | Imprecision | Heterogeneity | Incoherence | Confidence rating |
| --- | --- | --- | --- | --- | --- | --- | --- | --- |
| IBS-SSS | | | | | | | | |
| Face-to-face CBT:Digital CBT | 2 | Some concerns | Low risk | No concerns | No concerns | No concerns | No concerns | Moderate |
| Face-to-face CBT:Self-helped CBT | 2 | Some concerns | Low risk | No concerns | No concerns | Some concerns | No concerns | Low |
| Face-to-face CBT:Telephone-delivered CBT | 1 | Some concerns | Low risk | No concerns | No concerns | Some concerns | No concerns | Low |
| Face-to-face CBT:Alternative face-to-face psychotherapy | 0 | Major concerns | Low risk | Some concerns | Some concerns | No concerns | No concerns | Very low |
| IBS-QOL | | | | | | | | |
| Face-to-face CBT:Digital CBT | 2 | Some concerns | Low risk | No concerns | Some concerns | Some concerns | No concerns | Very low |
| Face-to-face CBT:Self-helped CBT | 1 | Some concerns | Low risk | No concerns | Some concerns | Some concerns | No concerns | Very low |
| Face-to-face CBT:Alternative face-to-face psychotherapy | 1 | Some concerns | Low risk | No concerns | No concerns | Some concerns | No concerns | Low |
| Face-to-face CBT:Alternative digital psychotherapy | 0 | Some concerns | Low risk | Some concerns | No concerns | Some concerns | No concerns | Very low |
| Face-to-face CBT:Alternative self-help psychotherapy | 0 | Some concerns | Low risk | Some concerns | Some concerns | No concerns | No concerns | Very low |
| API | | | | | | | | |
| Face-to-face CBT:Digital CBT | 1 | Some concerns | Low risk | No concerns | Some concerns | Some concerns | No concerns | Very low |

**Footnote:** CBT, cognitive behavioral therapy; IBS-SSS, irritable bowel syndrome symptom severity scale; IBS-QOL, irritable bowel syndrome quality of life; API, abdominal pain intensity.

**Table S7.** Value of SUCRA for each treatment on outcomes

| **Treatment** |  | **SUCRA** |  |
| --- | --- | --- | --- |
|  | **IBS-SSS** | **IBS-QOL** | **API** |
| Face-to-face CBT | 0.50 | 0.54 | 0.49 |
| Digital CBT | 0.53 | 0.71 | 0.54 |
| Telephone-delivered CBT | 0.53 | NA | NA |
| Self-help CBT | 0.56 | 0.64 | NA |
| Alternative face-to-face psychotherapy | 0.51 | 0.52 | NA |
| Alternative digital psychotherapy | NA | 0.51 | NA |
| Alternative self-help psychotherapy | NA | 0.52 | NA |

**Footnote:** SUCRA, surface under the cumulative rank curve; IBS-SSS, irritable bowel syndrome symptom severity scale; IBS-QOL, irritable bowel syndrome quality of life; API, abdominal pain intensity; CBT, cognitive behavioral therapy; NA, not applicable. Values nearest 1 indicate preferred treatment.

**Figure S1.** The “dev-dev” plots of random-effects consistency and inconsistency models for NMA by outcomes


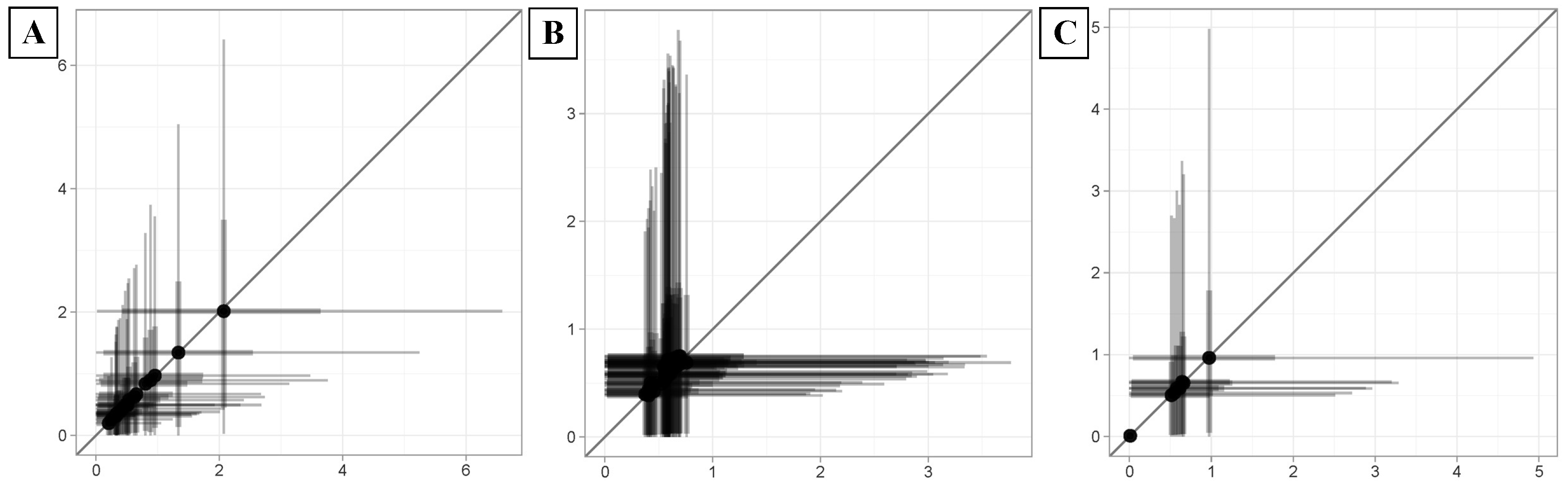


**Footnote:** NMA, network meta-analysis. A: IBS-SSS; B: IBS-QOL; C: API. The horizontal coordinate represents residual deviance of consistency model and the vertical coordinate represents residual deviance of inconsistency model. All points lie roughly on the line of equality, indicating that there is no evidence for inconsistency globally.

**Figure S2.** The node-splitting plots of the posterior distributions of the direct, indirect, and network estimation


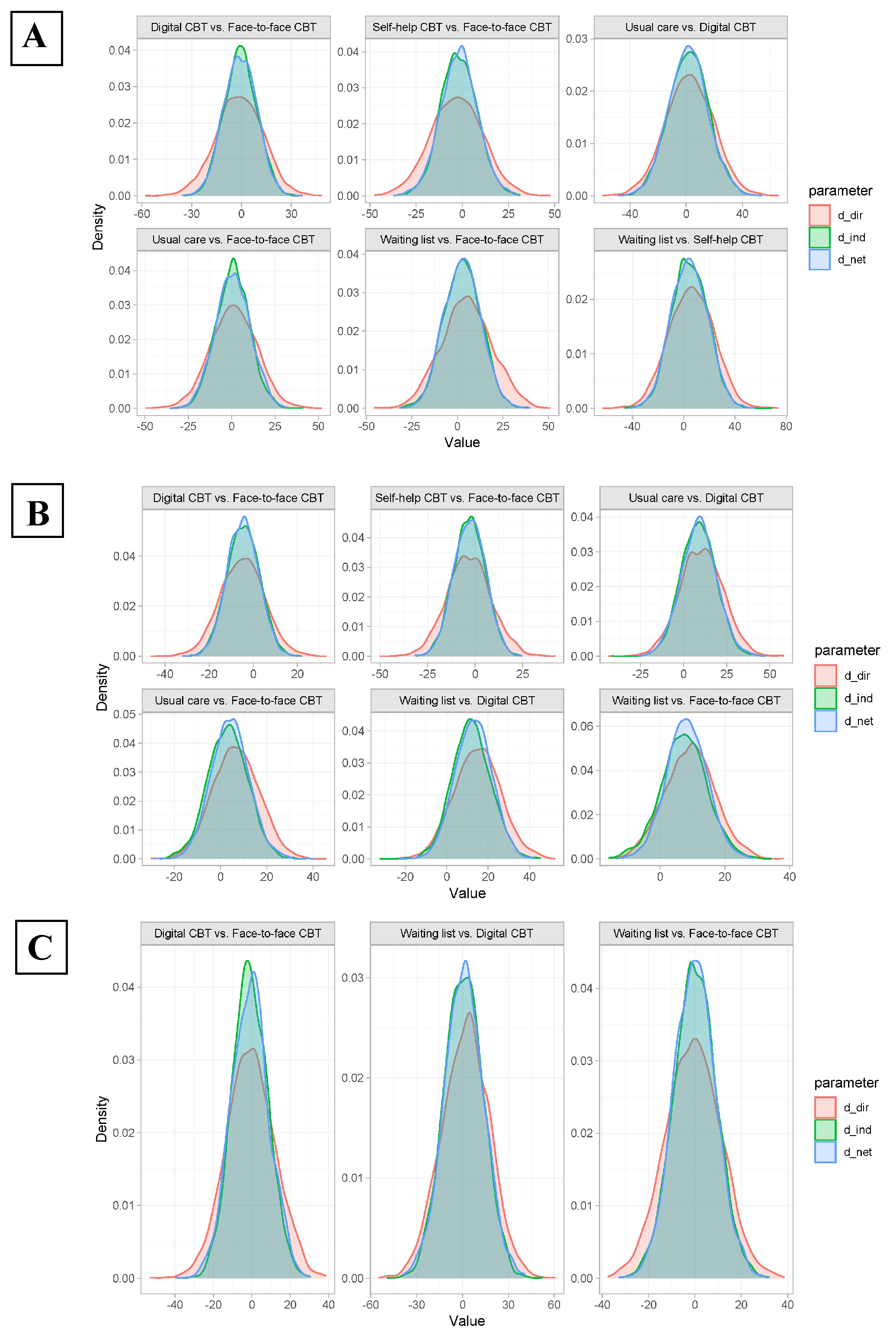


**Footnote:** A: IBS-SSS; B: IBS-QOL; C: API. The DIC of each inconsistency model is unchanged from the consistency model, no node-splits result in reduced heterogeneity standard deviation τ compared to the consistency model, and the Bayesian p-values more than 0.05. There is no evidence of inconsistency locally.

**Figure S3.** Network diagram of comparison of IBS-SSS


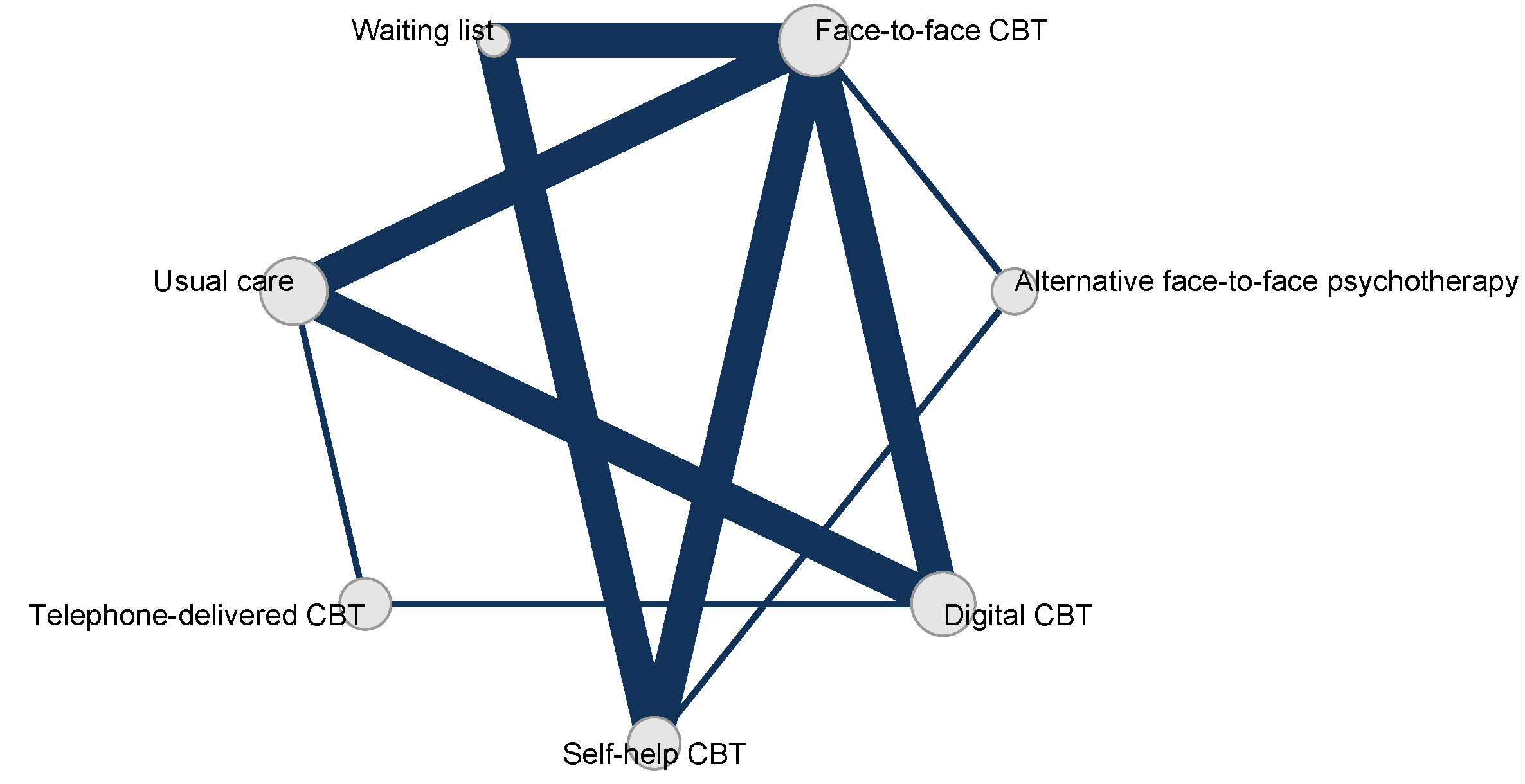


**Footnote:** IBS-SSS, irritable bowel syndrome symptom severity scale; CBT, cognitive behavioral therapy.

**Figure S4.** Sensitivity analysis of IBS-SSS after excluding RCTs at high risk of bias


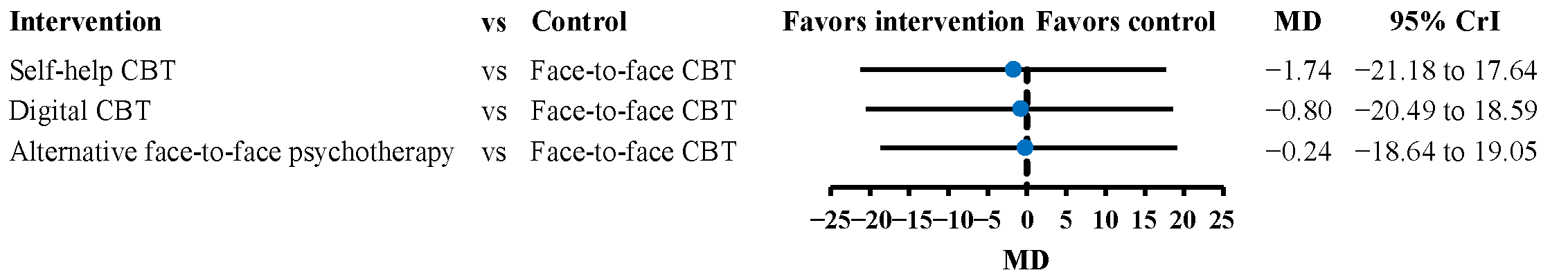


**Footnote**: IBS-SSS, irritable bowel syndrome symptom severity scale; RCTs, randomized controlled trials; CBT, cognitive behavioral therapy; MD, mean difference; CrI, credible interval. The black vertical line corresponds to 0.

**Figure S5.** Sensitivity analysis of IBS-SSS using frequentist methods


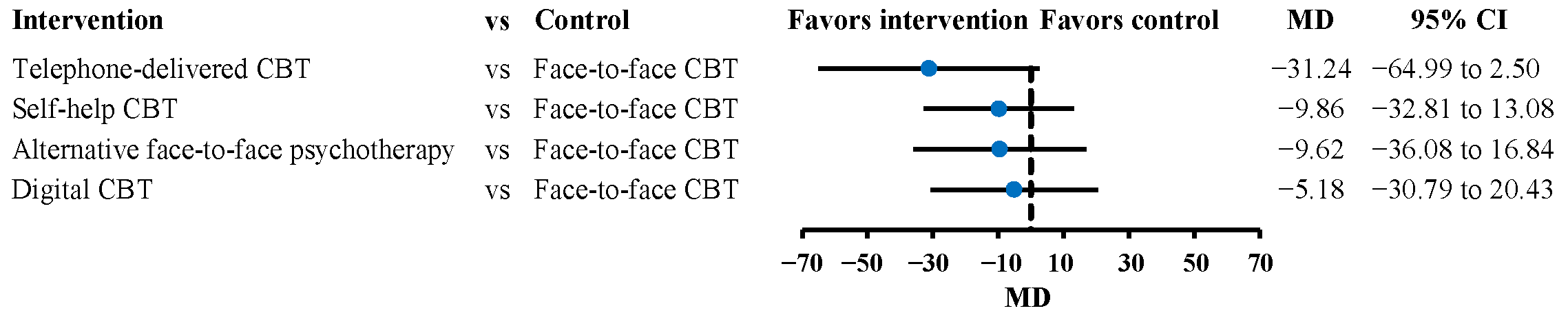


**Footnote**: IBS-SSS, irritable bowel syndrome symptom severity scale; RCTs, randomized controlled trials; CBT, cognitive behavioral therapy; MD, mean difference; CI, confidence interval. The black vertical line corresponds to 0.

**Figure S6.** Network diagram of comparison of IBS-QOL


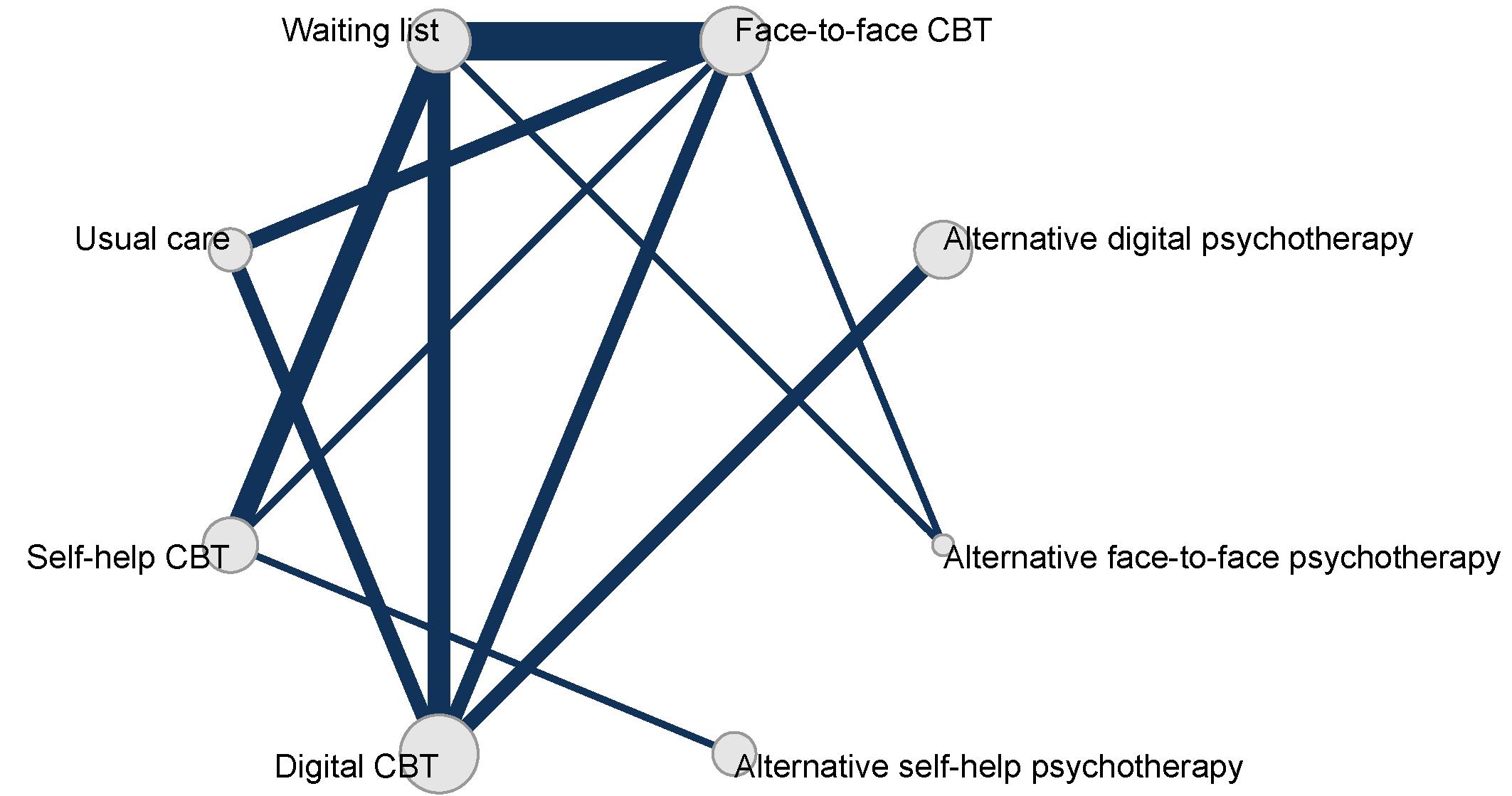


**Footnote:** IBS-QOL, irritable bowel syndrome quality of life; CBT, cognitive behavioral therapy.

**Figure S7.** Sensitivity analysis of IBS-QOL after excluding RCTs at high risk of bias


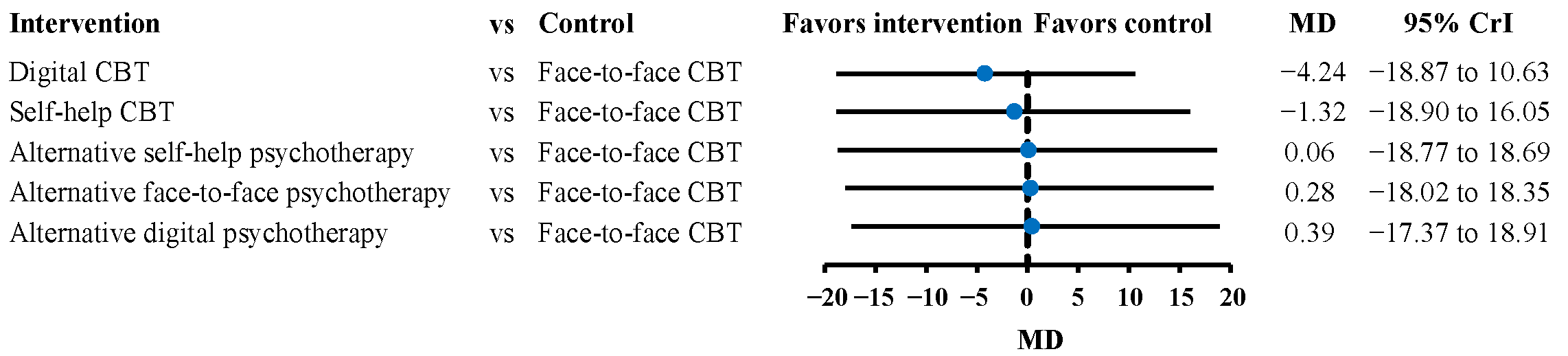


**Footnote**: IBS-QOL, irritable bowel syndrome quality of life; RCTs, randomized controlled trials; CBT, cognitive behavioral therapy; MD, mean difference; CrI, credible interval. The black vertical line corresponds to 0.

**Figure S8.** Sensitivity analysis of IBS-QOL using frequentist methods


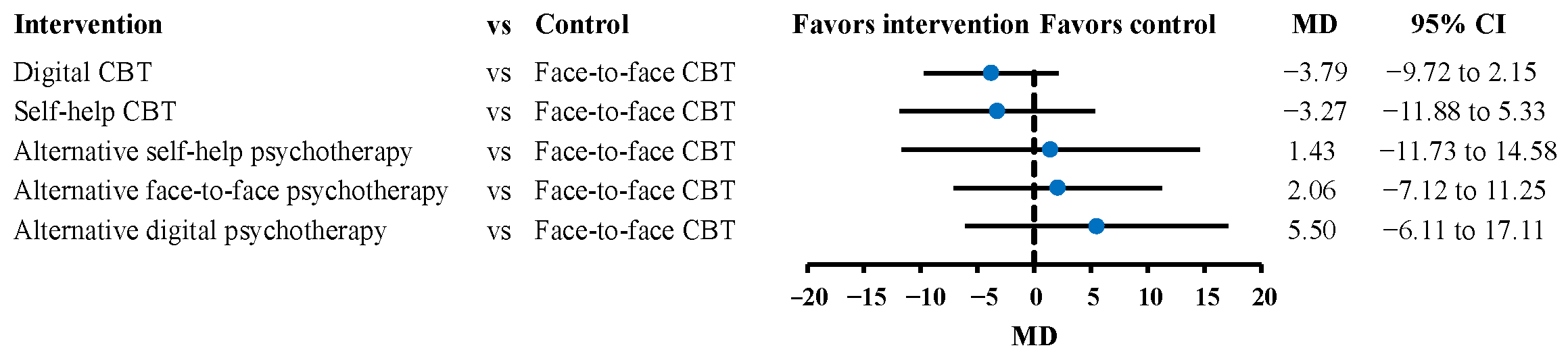


**Footnote**: IBS-QOL, irritable bowel syndrome quality of life; RCTs, randomized controlled trials; CBT, cognitive behavioral therapy; MD, mean difference; CI, confidence interval. The black vertical line corresponds to 0.

**Figure S9.** Network diagram of comparison of API


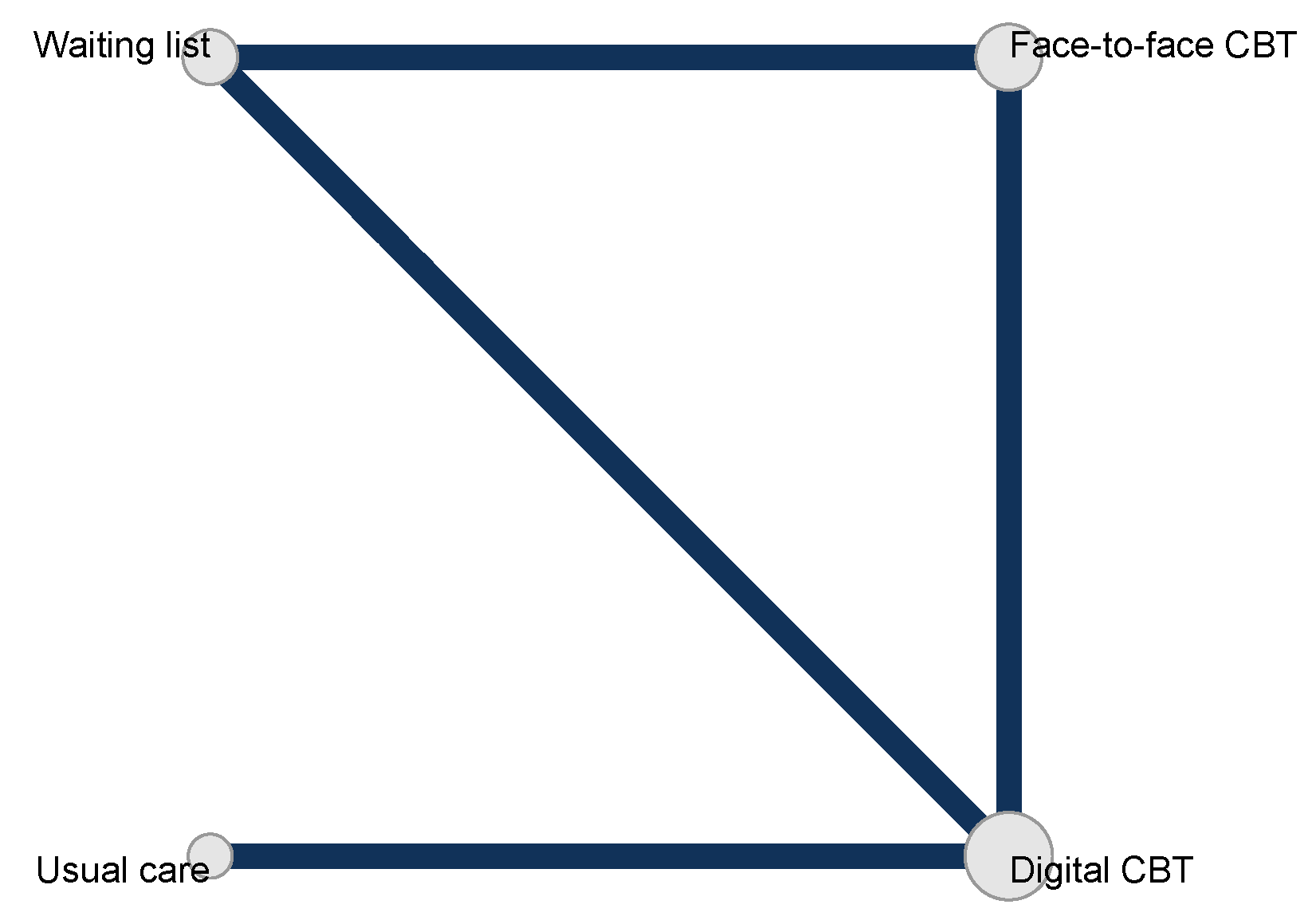


**Footnote:** API, abdominal pain intensity; CBT, cognitive behavioral therapy.

**Figure S10.** Effect of comparison between face-to-face CBT and digital CBT of API


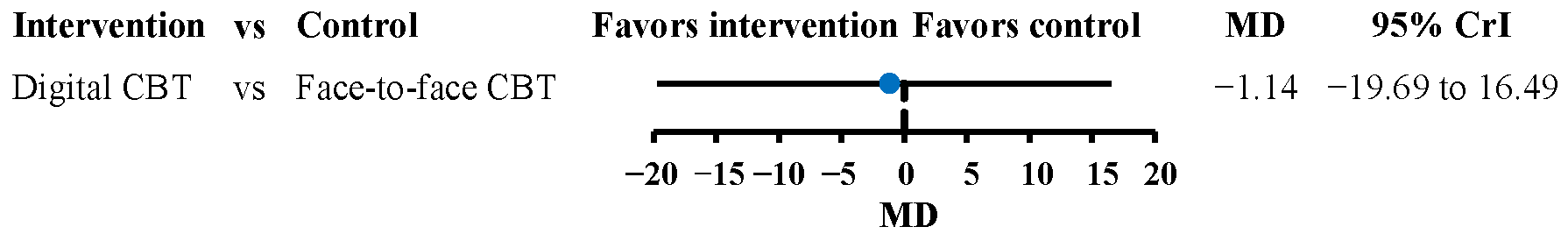


**Footnote**: CBT, cognitive behavioral therapy; API, abdominal pain intensity; MD, mean difference; CrI, credible interval. The black vertical line corresponds to 0.

**Figure S11.** Sensitivity analysis of API using frequentist methods


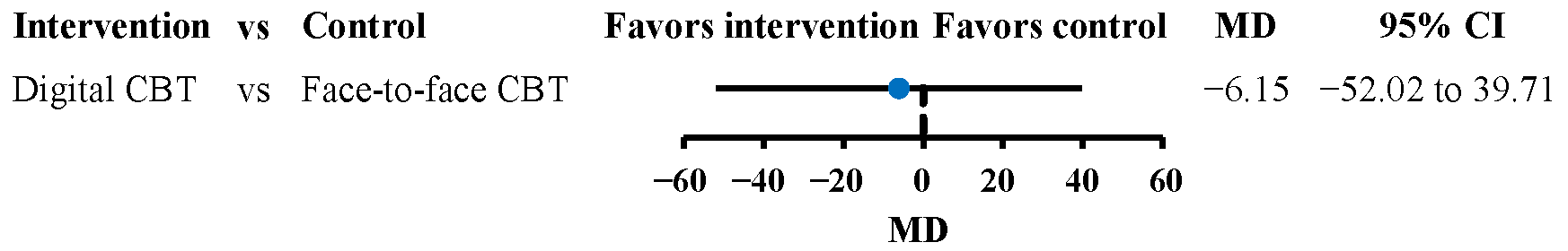


**Footnote**: API, abdominal pain intensity; CBT, cognitive behavioral therapy; MD, mean difference; CI, confidence interval. The black vertical line corresponds to 0.

**Figure S12.** Subgroup analysis of IBS-SSS on treatment duration


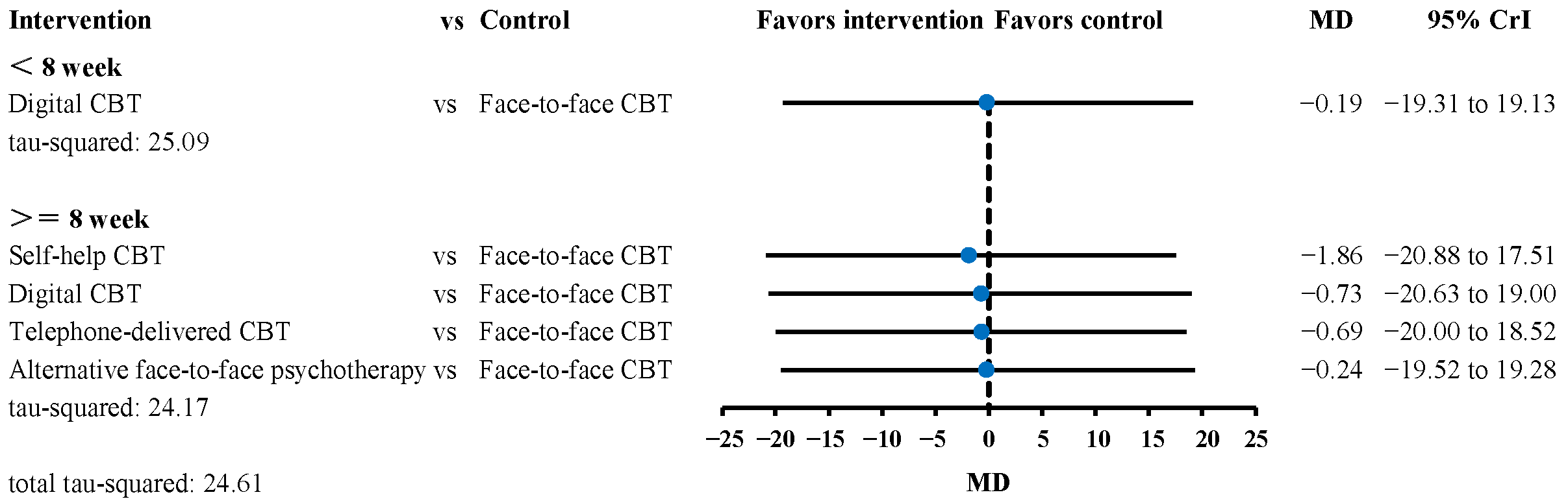


**Footnote**: IBS-SSS, irritable bowel syndrome symptom severity scale; CBT, cognitive behavioral therapy; MD, mean difference; CrI, credible interval.

**Figure S13.** Subgroup analysis of IBS-SSS on delivery method of face-to-face CBT


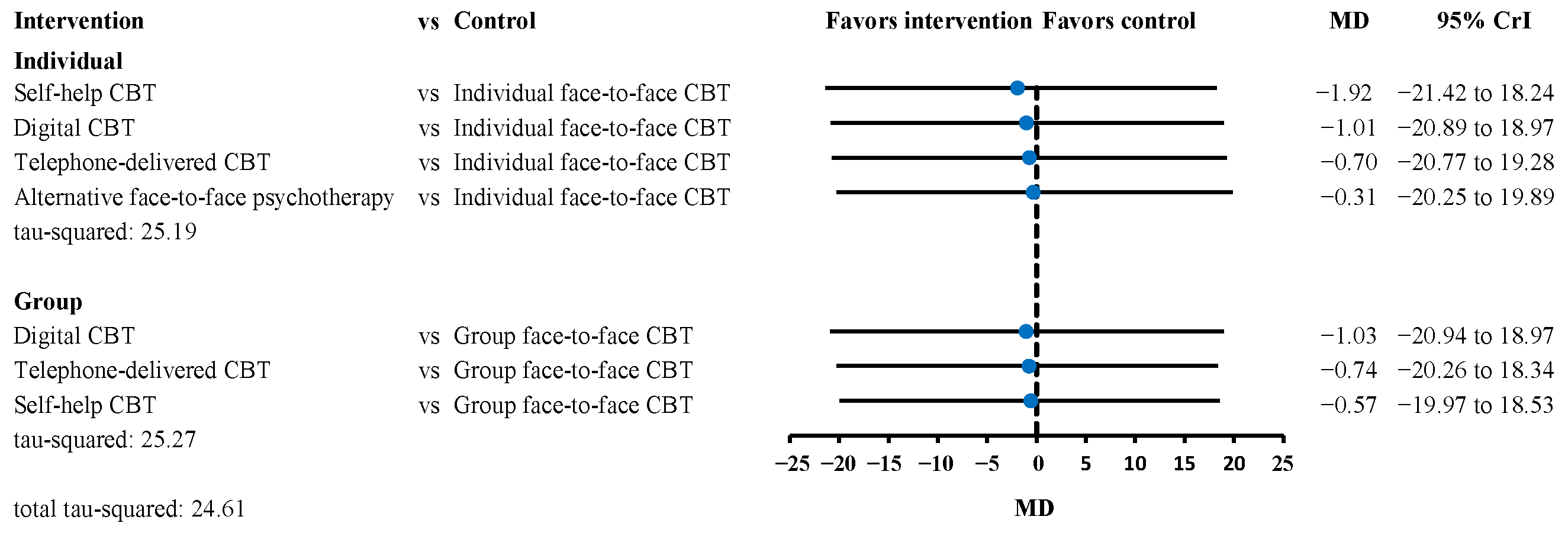


**Footnote**: IBS-SSS, irritable bowel syndrome symptom severity scale; CBT, cognitive behavioral therapy; MD, mean difference; CrI, credible interval.

**Figure S14.** Subgroup analysis of IBS-SSS on the guidance level of self-help CBT


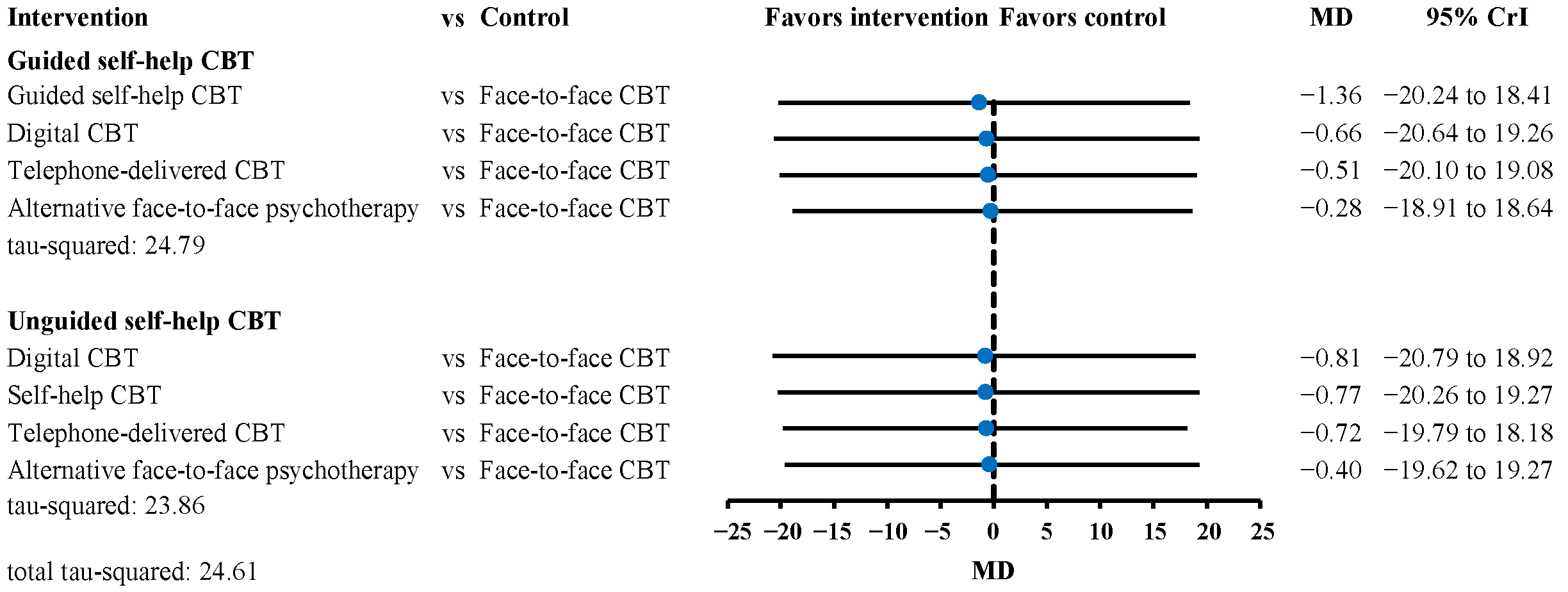


**Footnote**: IBS-SSS, irritable bowel syndrome symptom severity scale; CBT, cognitive behavioral therapy; MD, mean difference; CrI, credible interval.
