# Supplementary material for: Face-to-Face Versus Digital, Telephone-Delivered, and Self-Help Cognitive Behavioral Therapy for Irritable Bowel Syndrome: Systematic Review and Bayesian Indirect Treatment Comparison Meta-Analysis
Source: J Med Internet Res. 2026 Jan 8;28:e75833. doi: 10.2196/75833 (PMC12782461; doi:10.2196/75833)
Supplement: Multimedia Appendix 2 [file jmir-v28-e75833-s002.docx]

**Supplementary materials files**

eTable 1. Search strategy in Ovid Medline 2

eTable 2. Search strategy in Embase 3

eTable 3. Search strategy in Cochrane Library 4

eTable 4. Definition of different CBT 5

eTable 5. Summary of fixed-effects and random-effects model fit statistics from network meta-analysis 6

eTable 6. Certainty of evidence of outcomes 7

eTable 7. Value of SUCRA for each treatment on outcomes 9

eFigure 1. The “dev-dev” plots of random-effects consistency and inconsistency models for NMA by outcomes 10

eFigure 2. The node-splitting plots of the posterior distributions of the direct, indirect, and network estimation 11

eFigure 3. Network diagram of comparison of IBS-SSS 12

eFigure 4. Sensitivity analysis of IBS-SSS after excluding RCTs at high risk of bias 13

eFigure 5. Sensitivity analysis of IBS-SSS using frequentist methods 14

eFigure 6. Network diagram of comparison of IBS-QOL 15

eFigure 7. Sensitivity analysis of IBS-QOL after excluding RCTs at high risk of bias 16

eFigure 8. Sensitivity analysis of IBS-QOL using frequentist methods 17

eFigure 9. Network diagram of comparison of API 18

eFigure 10. Effect of comparison between face-to-face CBT and digital CBT of API 19

eFigure 11. Sensitivity analysis of API using frequentist methods 20

eFigure 12. Subgroup analysis of IBS-SSS on treatment duration 21

eFigure 13. Subgroup analysis of IBS-SSS on delivery method of face-to-face CBT 22

eFigure 14. Subgroup analysis of IBS-SSS on the guidance level of self-help CBT 23

**eTable 1.** Search strategy in Ovid Medline

| **ID** | **Search strategy** |
| --- | --- |
| 1 | randomized controlled trial.pt. |
| 2 | controlled clinical trial.pt. |
| 3 | randomized.ab,ti. |
| 4 | randomised.ab,ti. |
| 5 | randomly.ab,ti. |
| 6 | trial.ab,ti. |
| 7 | groups.ab,ti. |
| 8 | 1 or 2 or 3 or 4 or 5 or 6 or 7 |
| 9 | limit 8 to humans |
| 10 | exp irritable bowel syndrome/ |
| 11 | (irritable bowel syndrome or IBS).ab,ti. |
| 12 | 11 or 12 |
| 13 | exp cognitive behavioral therapy/ |
| 14 | (cognitive behavioural therapy or cognitive behavioral therapy or CBT or behavioural therapy or behavioral therapy).ab,ti. |
| 15 | 13 or 14 |
| 16 | 9 and 12 and 15 |

**eTable 2.** Search strategy in Embase

| **ID** | **Search strategy** |
| --- | --- |
| 1 | 'randomized controlled trial'/exp |
| 2 | 'controlled clinical trial'/exp |
| 3 | randomized:ab,ti |
| 4 | randomised:ab,ti |
| 5 | randomly:ab,ti |
| 6 | trial:ab,ti |
| 7 | groups:ab,ti |
| 8 | #1 OR #2 OR #3 OR #4 OR #5 OR #6 OR #7 |
| 9 | (#1 OR #2 OR #3 OR #4 OR #5 OR #6 OR #7) AND [humans]/lim |
| 10 | 'irritable bowel syndrome'/exp |
| 11 | 'irritable bowel syndrome':ab,ti OR IBS:ab,ti |
| 12 | #10 OR #11 |
| 13 | 'cognitive behavioural therapy'/exp |
| 14 | 'cognitive behavioral therapy'/exp |
| 15 | CBT/exp |
| 16 | 'cognitive behavioural therapy':ab,ti OR 'cognitive behavioral therapy':ab,ti OR CBT:ab,ti OR 'behavioural therapy':ab,ti OR 'behavioral therapy':ab,ti |
| 17 | #13 OR #14 OR #15 OR #16 |
| 18 | #9 AND #12 AND #17 |

**eTable 3.** Search strategy in Cochrane Library

| **ID** | **Search strategy** |
| --- | --- |
| 1 | (randomized controlled trial):pt |
| 2 | (controlled clinical trial):pt |
| 3 | (randomized):ti,ab,kw |
| 4 | (randomised):ti,ab,kw |
| 5 | (randomly):ti,ab,kw |
| 6 | (trial):ti,ab,kw |
| 7 | (groups):ti,ab,kw |
| 8 | #1 OR #2 OR #3 OR #4 OR #5 OR #6 OR #7 in Trials |
| 9 | MeSH descriptor: [irritable bowel syndrome] explode all trees |
| 10 | (irritable bowel syndrome OR IBS):ti,ab,kw |
| 11 | #9 OR #10 |
| 12 | MeSH descriptor: [cognitive behavioral therapy] explode all trees |
| 13 | (cognitive behavioural therapy OR cognitive behavioral therapy OR CBT OR behavioural therapy OR behavioural therapy):ti,ab,kw |
| 14 | #12 OR #13 |
| 15 | #8 AND #11 AND #14 |

**eTable 4.** Definition of different CBT

| **Interventions** | **Definitions** |
| --- | --- |
| Face-to-face CBT | CBT is delivered face-to-face in clinics via scheduled, therapist-guided sessions conducted individually or in groups. |
| Digital CBT | CBT is delivered through web or mobile applications with therapist guidance. |
| Telephone-delivered CBT | CBT is delivered through telephone with therapist guidance. |
| Self-help CBT | CBT delivery relies mainly on structured written or web-based self-help materials, which may be therapist-guided or unguided. |

**Footnote:** CBT, cognitive behavioral therapy.

**eTable 5.** Summary of fixed-effects and random-effects model fit statistics from network meta-analysis

| **Model** | **Number of data points** | **Posterior total residual deviance** | **DIC** |
| --- | --- | --- | --- |
| **IBS-SSS** | | | |
| **FE consistency** | 20 | 12.2 | 18.6 |
| **FE inconsistency** | 20 | 12.5 | 19.1 |
| **RE consistency** | 20 | 12.3 | 18.6 |
| **IBS-QOL** | | | |
| **FE consistency** | 35 | 20.2 | 39.7 |
| **FE inconsistency** | 35 | 22.1 | 41.1 |
| **RE consistency** | 35 | 20.8 | 40 |
| **API** | | | |
| **FE consistency** | 8 | 4.5 | 8.7 |
| **FE inconsistency** | 8 | 4.6 | 8.9 |
| **RE consistency** | 8 | 4.5 | 8.7 |

**Footnote:** DIC, deviance information criteria; IBS-SSS, irritable bowel syndrome symptom severity scale; FE, fixed-effect; RE, random-effect; IBS-QOL, irritable bowel syndrome quality of life; API, abdominal pain intensity.

**eTable 6.** Certainty of evidence of outcomes

| Comparison | Number of studies | Within-study bias | Reporting bias | Indirectness | Imprecision | Heterogeneity | Incoherence | Confidence rating |
| --- | --- | --- | --- | --- | --- | --- | --- | --- |
| IBS-SSS | | | | | | | | |
| Face-to-face CBT:Digital CBT | 2 | Some concerns | Low risk | No concerns | No concerns | No concerns | No concerns | Moderate |
| Face-to-face CBT:Self-helped CBT | 2 | Some concerns | Low risk | No concerns | No concerns | Some concerns | No concerns | Low |
| Face-to-face CBT:Telephone-delivered CBT | 1 | Some concerns | Low risk | No concerns | No concerns | Some concerns | No concerns | Low |
| Face-to-face CBT:Alternative face-to-face psychotherapy | 0 | Major concerns | Low risk | Some concerns | Some concerns | No concerns | No concerns | Very low |
| IBS-QOL | | | | | | | | |
| Face-to-face CBT:Digital CBT | 2 | Some concerns | Low risk | No concerns | Some concerns | Some concerns | No concerns | Very low |
| Face-to-face CBT:Self-helped CBT | 1 | Some concerns | Low risk | No concerns | Some concerns | Some concerns | No concerns | Very low |
| Face-to-face CBT:Alternative face-to-face psychotherapy | 1 | Some concerns | Low risk | No concerns | No concerns | Some concerns | No concerns | Low |
| Face-to-face CBT:Alternative digital psychotherapy | 0 | Some concerns | Low risk | Some concerns | No concerns | Some concerns | No concerns | Very low |
| Face-to-face CBT:Alternative self-help psychotherapy | 0 | Some concerns | Low risk | Some concerns | Some concerns | No concerns | No concerns | Very low |
| API | | | | | | | | |
| Face-to-face CBT:Digital CBT | 1 | Some concerns | Low risk | No concerns | Some concerns | Some concerns | No concerns | Very low |

**Footnote:** CBT, cognitive behavioral therapy; IBS-SSS, irritable bowel syndrome symptom severity scale; IBS-QOL, irritable bowel syndrome quality of life; API, abdominal pain intensity.

**eTable 7.** Value of SUCRA for each treatment on outcomes

| **Treatment** |  | **SUCRA** |  |
| --- | --- | --- | --- |
|  | **IBS-SSS** | **IBS-QOL** | **API** |
| Face-to-face CBT | 0.50 | 0.54 | 0.49 |
| Digital CBT | 0.53 | 0.71 | 0.54 |
| Telephone-delivered CBT | 0.53 | NA | NA |
| Self-help CBT | 0.56 | 0.64 | NA |
| Alternative face-to-face psychotherapy | 0.51 | 0.52 | NA |
| Alternative digital psychotherapy | NA | 0.51 | NA |
| Alternative self-help psychotherapy | NA | 0.52 | NA |

**Footnote:** SUCRA, surface under the cumulative rank curve; IBS-SSS, irritable bowel syndrome symptom severity scale; IBS-QOL, irritable bowel syndrome quality of life; API, abdominal pain intensity; CBT, cognitive behavioral therapy; NA, not applicable. Values nearest 1 indicate preferred treatment.

**eFigure 1.** The “dev-dev” plots of random-effects consistency and inconsistency models for NMA by outcomes


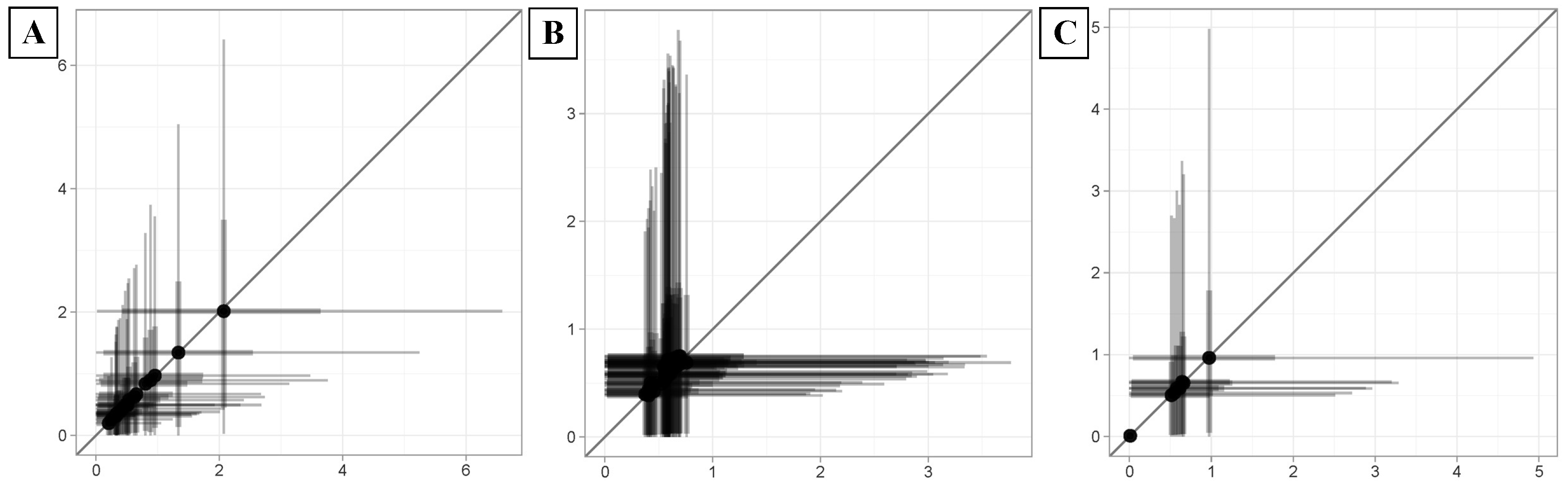


**Footnote:** NMA, network meta-analysis. A: IBS-SSS; B: IBS-QOL; C: API. The horizontal coordinate represents residual deviance of consistency model and the vertical coordinate represents residual deviance of inconsistency model. All points lie roughly on the line of equality, indicating that there is no evidence for inconsistency globally.

**eFigure 2.** The node-splitting plots of the posterior distributions of the direct, indirect, and network estimation


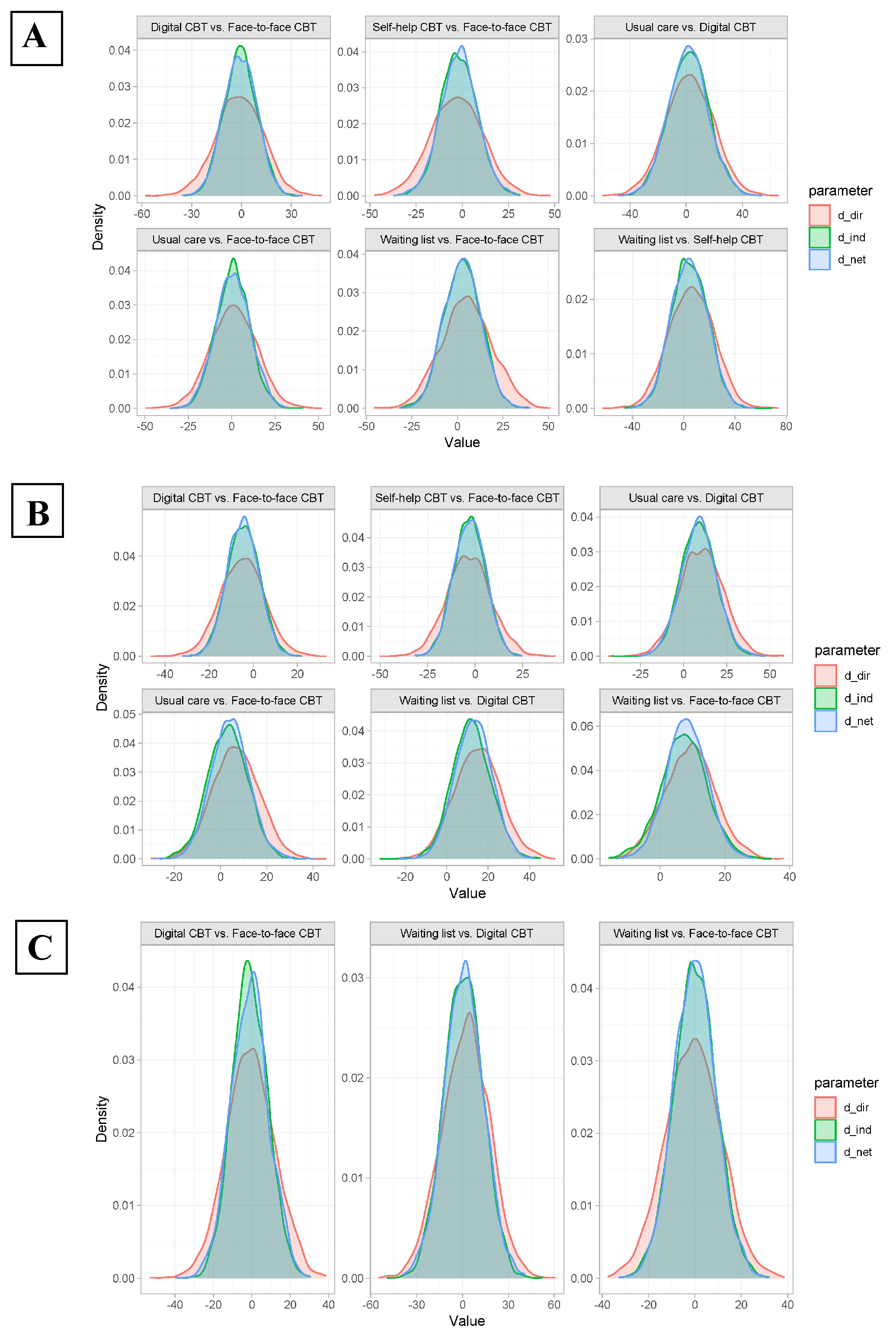


**Footnote:** A: IBS-SSS; B: IBS-QOL; C: API. The DIC of each inconsistency model is unchanged from the consistency model, no node-splits result in reduced heterogeneity standard deviation τ compared to the consistency model, and the Bayesian p-values more than 0.05. There is no evidence of inconsistency locally.

**eFigure 3.** Network diagram of comparison of IBS-SSS


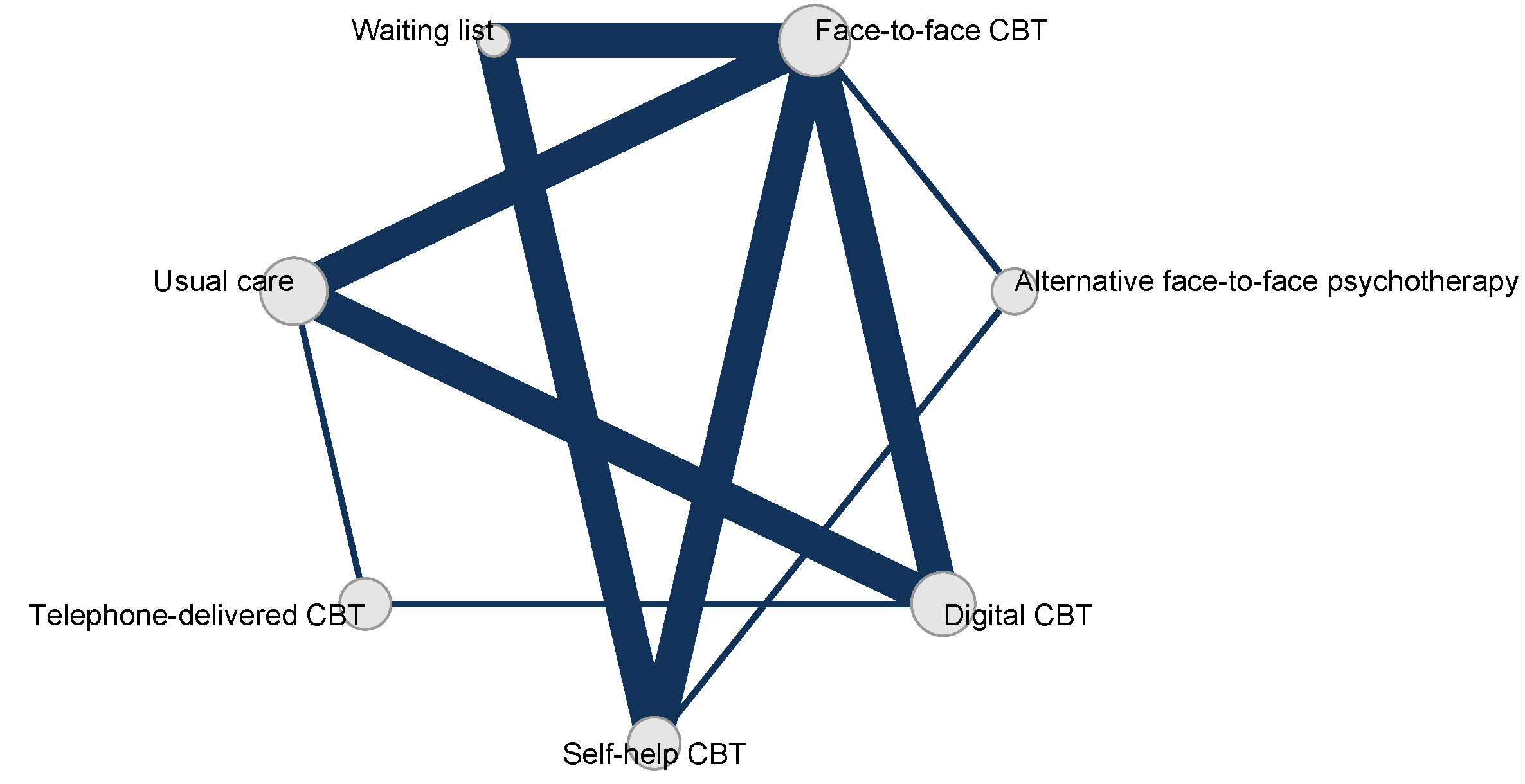


**Footnote:** IBS-SSS, irritable bowel syndrome symptom severity scale; CBT, cognitive behavioral therapy.

**eFigure 4.** Sensitivity analysis of IBS-SSS after excluding RCTs at high risk of bias


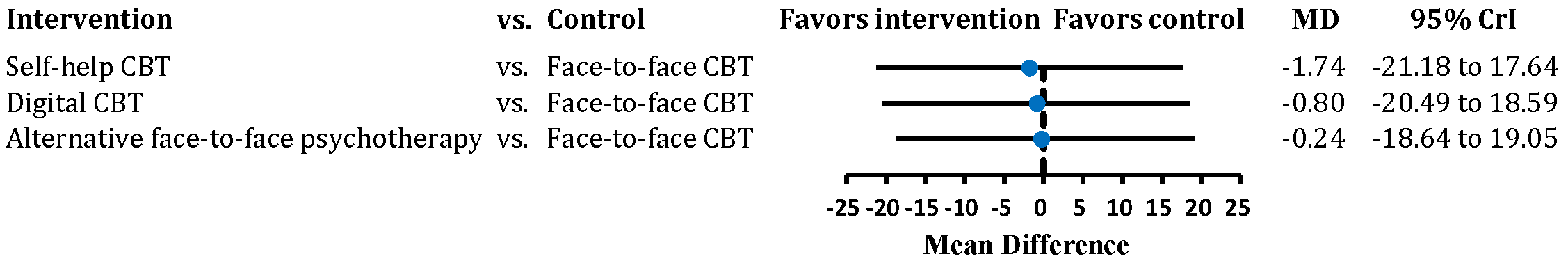


**Footnote**: IBS-SSS, irritable bowel syndrome symptom severity scale; RCTs, randomized controlled trials; CBT, cognitive behavioral therapy; MD, mean difference; CrI, credible interval. The black vertical line corresponds to 0.

**eFigure 5.** Sensitivity analysis of IBS-SSS using frequentist methods


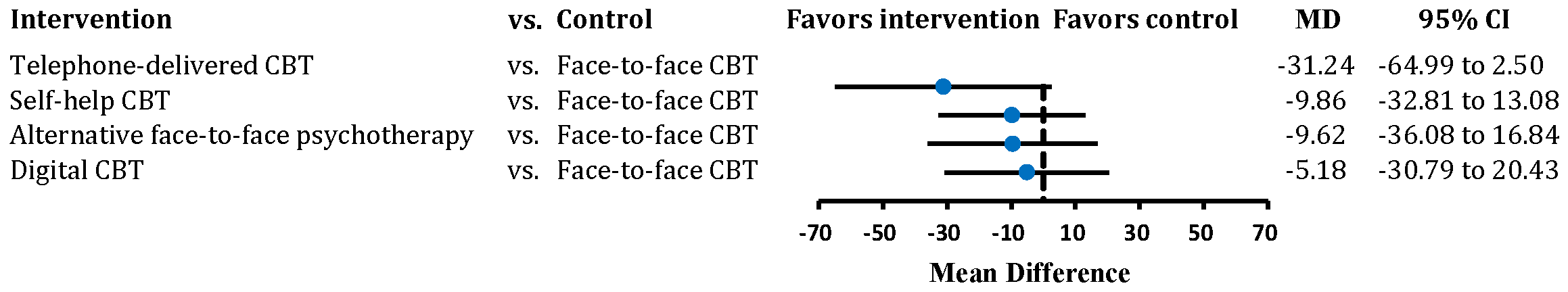


**Footnote**: IBS-SSS, irritable bowel syndrome symptom severity scale; RCTs, randomized controlled trials; CBT, cognitive behavioral therapy; MD, mean difference; CI, confidence interval. The black vertical line corresponds to 0.

**eFigure 6.** Network diagram of comparison of IBS-QOL


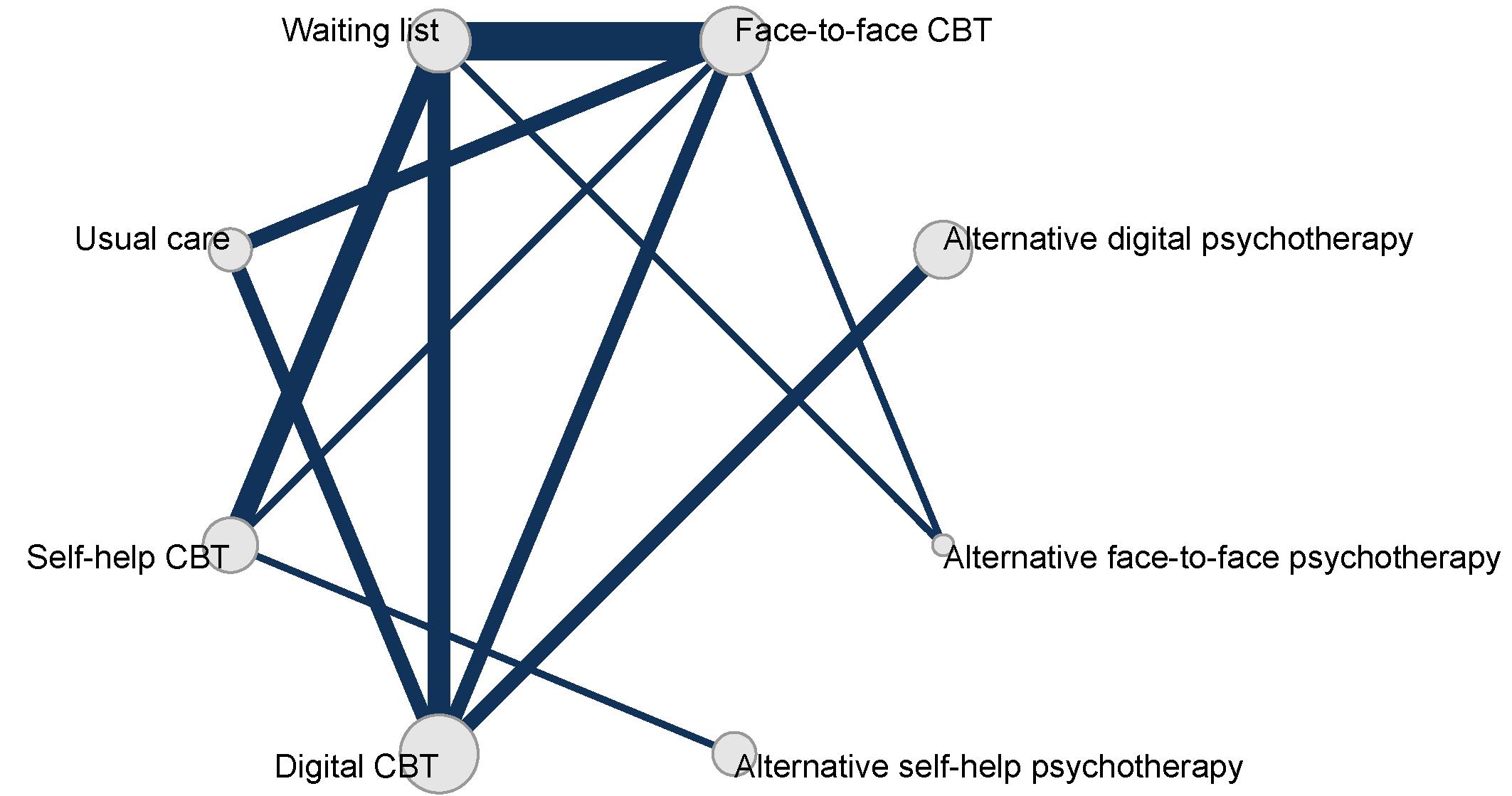


**Footnote:** IBS-QOL, irritable bowel syndrome quality of life; CBT, cognitive behavioral therapy.

**eFigure 7.** Sensitivity analysis of IBS-QOL after excluding RCTs at high risk of bias


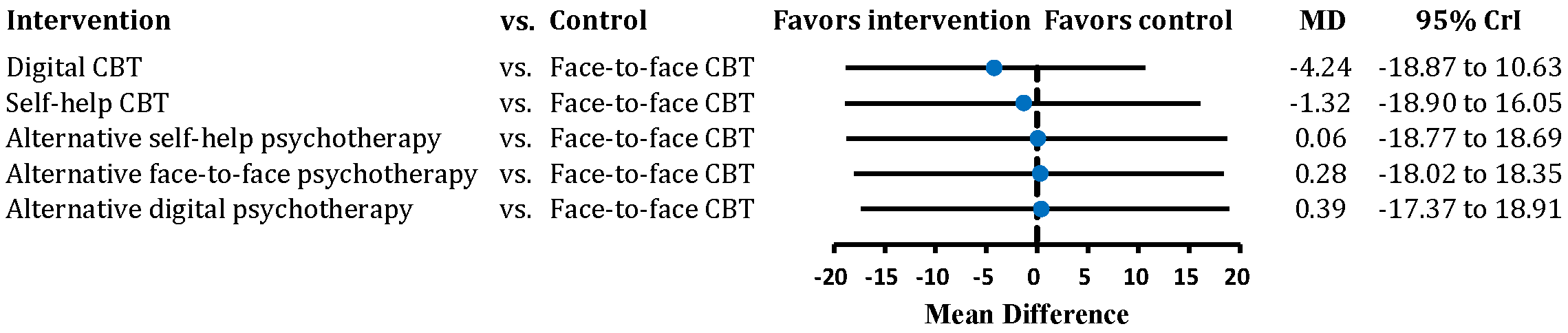


**Footnote**: IBS-QOL, irritable bowel syndrome quality of life; RCTs, randomized controlled trials; CBT, cognitive behavioral therapy; MD, mean difference; CrI, credible interval. The black vertical line corresponds to 0.

**eFigure 8.** Sensitivity analysis of IBS-QOL using frequentist methods


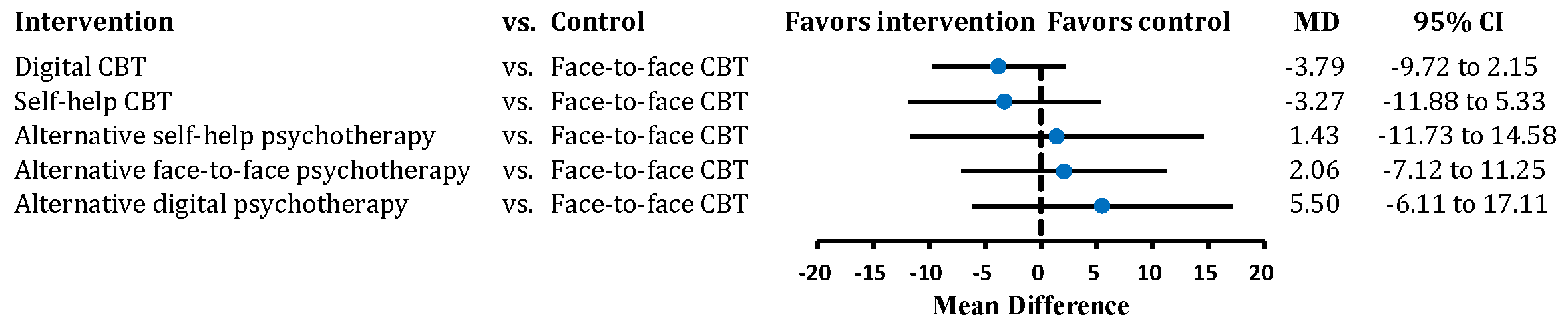


**Footnote**: IBS-QOL, irritable bowel syndrome quality of life; RCTs, randomized controlled trials; CBT, cognitive behavioral therapy; MD, mean difference; CI, confidence interval. The black vertical line corresponds to 0.

**eFigure 9.** Network diagram of comparison of API


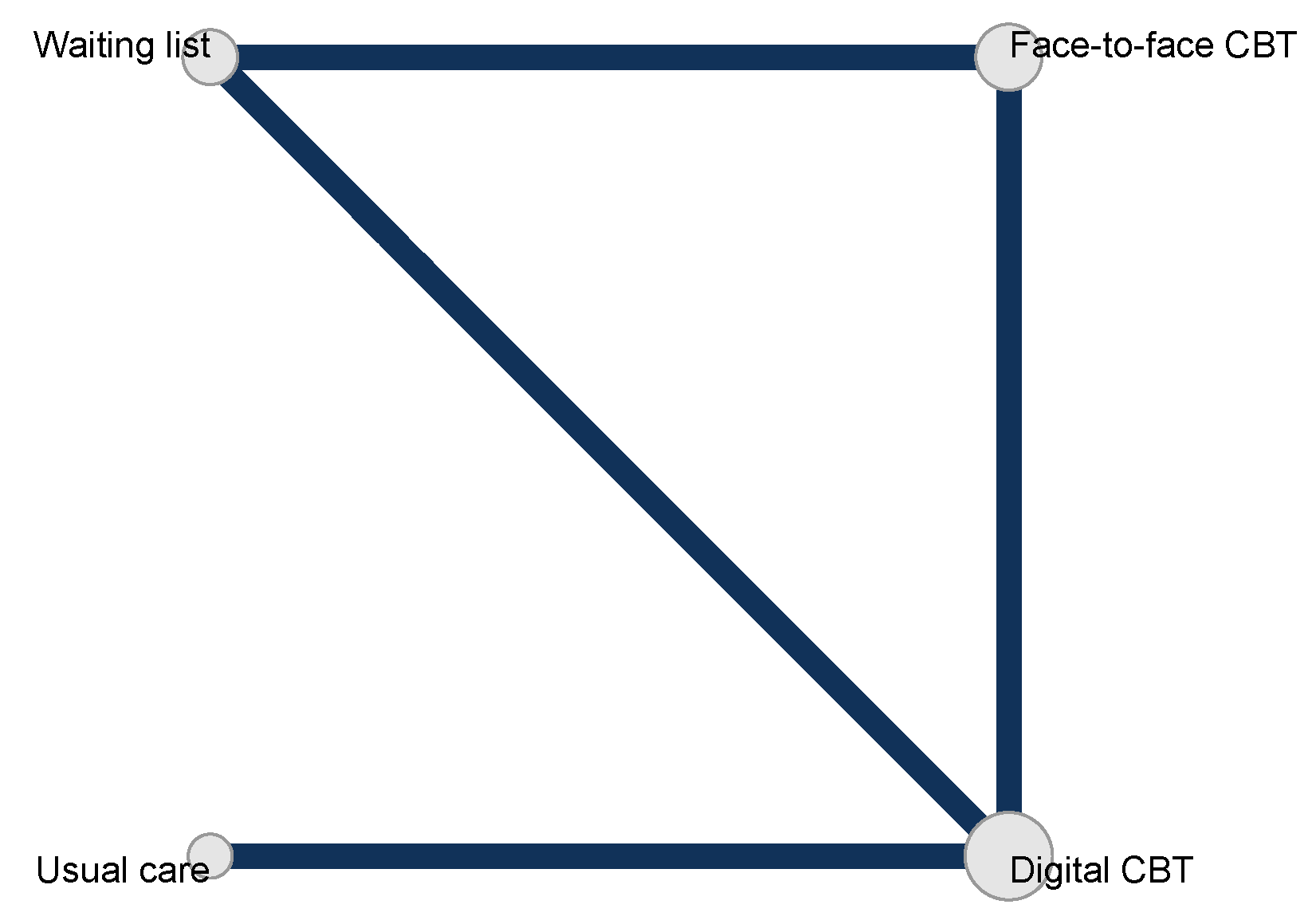


**Footnote:** API, abdominal pain intensity; CBT, cognitive behavioral therapy.

**eFigure 10.** Effect of comparison between face-to-face CBT and digital CBT of API


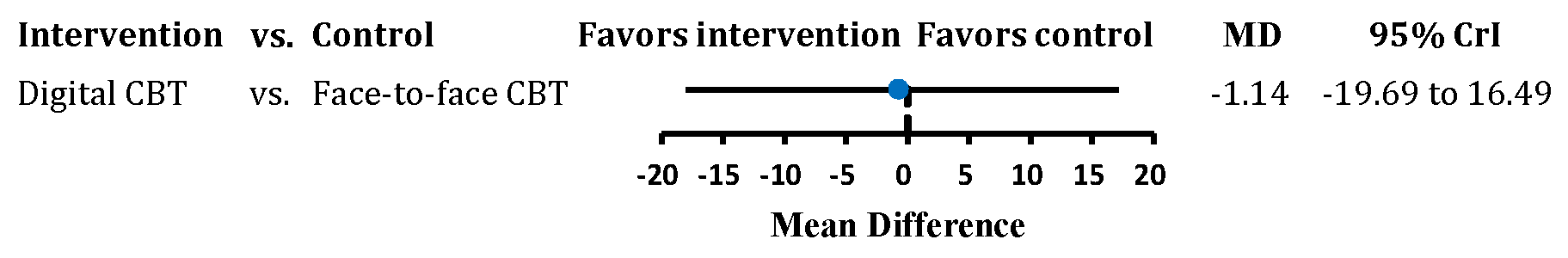


**Footnote**: CBT, cognitive behavioral therapy; API, abdominal pain intensity; MD, mean difference; CrI, credible interval. The black vertical line corresponds to 0.

**eFigure 11.** Sensitivity analysis of API using frequentist methods


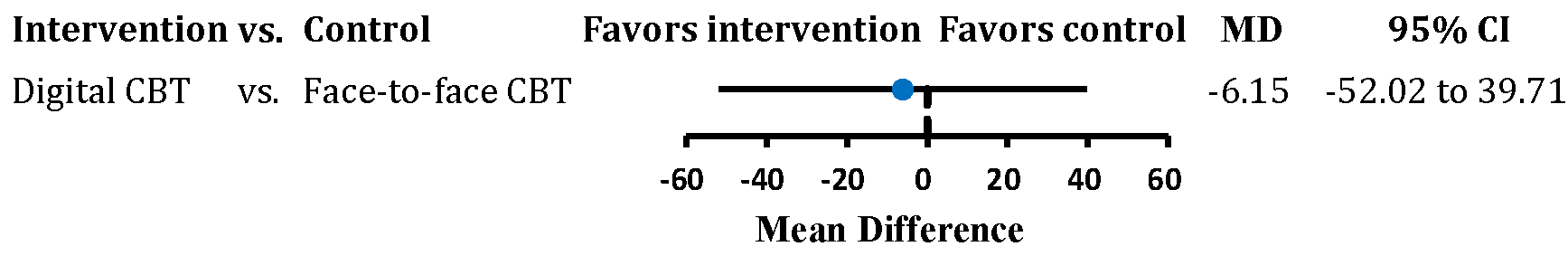


**Footnote**: API, abdominal pain intensity; CBT, cognitive behavioral therapy; MD, mean difference; CI, confidence interval. The black vertical line corresponds to 0.

**eFigure 12.** Subgroup analysis of IBS-SSS on treatment duration


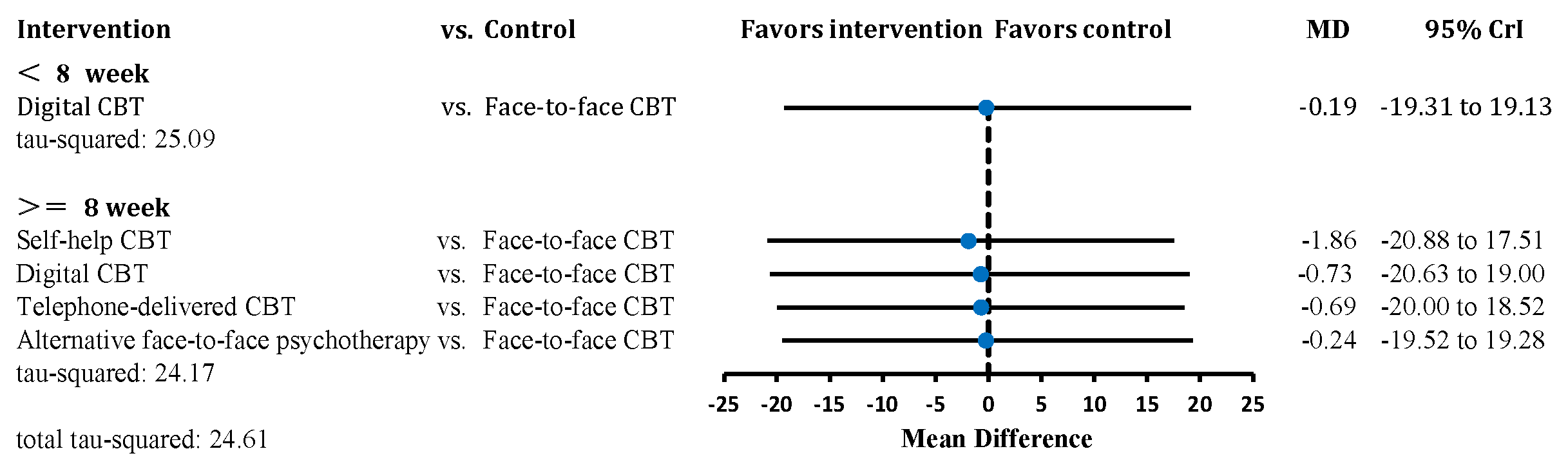


**Footnote**: IBS-SSS, irritable bowel syndrome symptom severity scale; CBT, cognitive behavioral therapy; MD, mean difference; CrI, credible interval.

**eFigure 13.** Subgroup analysis of IBS-SSS on delivery method of face-to-face CBT


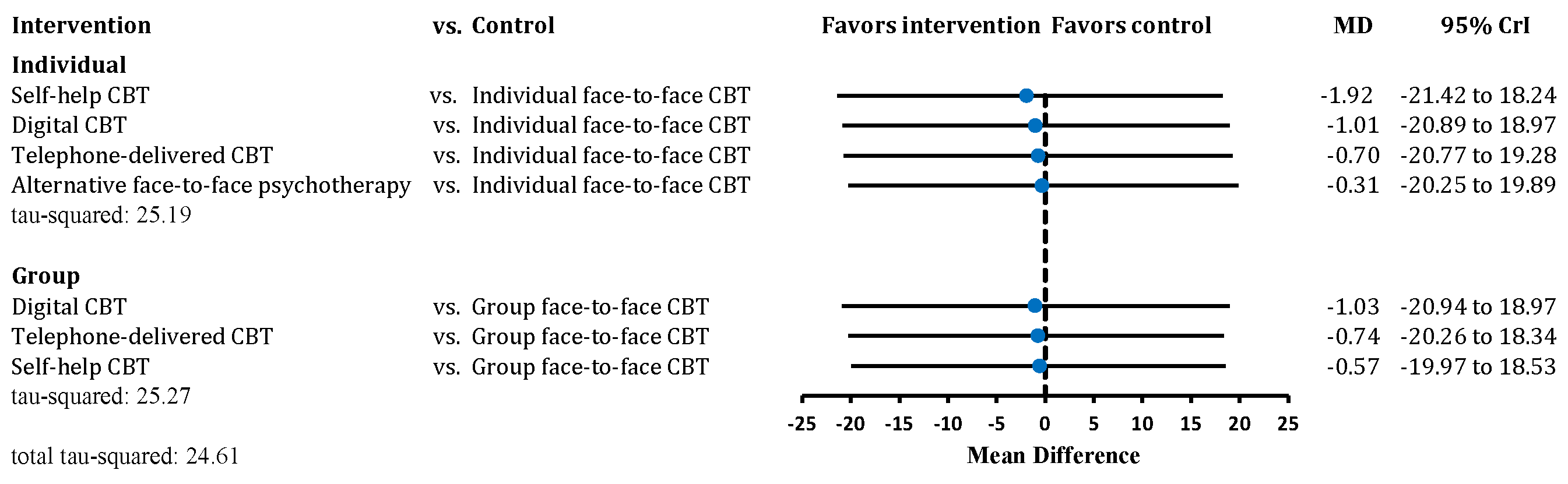


**Footnote**: IBS-SSS, irritable bowel syndrome symptom severity scale; CBT, cognitive behavioral therapy; MD, mean difference; CrI, credible interval.

**eFigure 14.** Subgroup analysis of IBS-SSS on the guidance level of self-help CBT


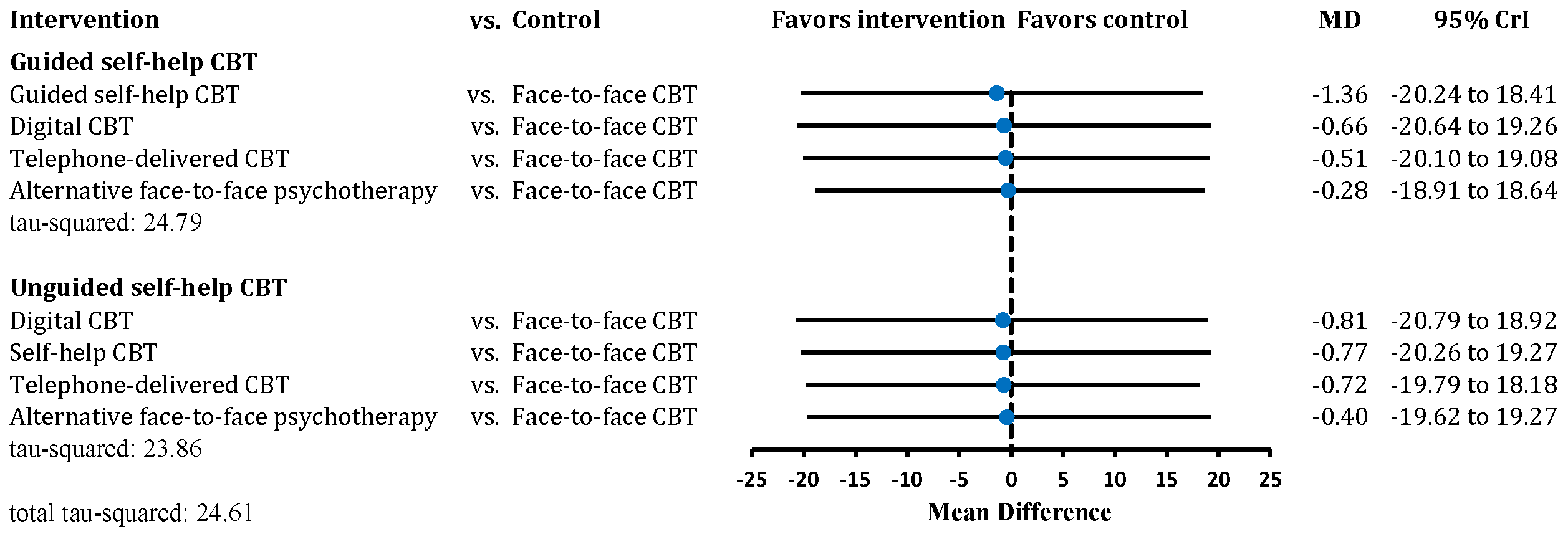


**Footnote**: IBS-SSS, irritable bowel syndrome symptom severity scale; CBT, cognitive behavioral therapy; MD, mean difference; CrI, credible interval.
