# Supplementary material for: Face-to-Face Versus Digital, Telephone-Delivered, and Self-Help Cognitive Behavioral Therapy for Irritable Bowel Syndrome: Systematic Review and Bayesian Indirect Treatment Comparison Meta-Analysis
Source: J Med Internet Res. 2026 Jan 8;28:e75833. doi: 10.2196/75833 (PMC12782461; doi:10.2196/75833)
Supplement: Checklist 1 [file jmir-v28-e75833-s003.pdf]

PRISMA-S Checklist

Section/topic	#	Checklist item	Location(s) Reported
**INFORMATION SOURCES AND METHODS**			
Database name	1	Name each individual database searched, stating the platform for each.	P9
Multi-database searching	2	If databases were searched simultaneously on a single platform, state the name of the platform, listing all of the databases searched.	P9
Study registries	3	List any study registries searched.	P8
Online resources and browsing	4	Describe any online or print source purposefully searched or browsed (e.g., tables of contents, print conference proceedings, web sites), and how this was done.	P10
Citation searching	5	Indicate whether cited references or citing references were examined, and describe any methods used for locating cited/citing references (e.g., browsing reference lists, using a citation index, setting up email alerts for references citing included studies).	P10
Contacts	6	Indicate whether additional studies or data were sought by contacting authors, experts, manufacturers, or others.	P10
Other methods	7	Describe any additional information sources or search methods used.	P10
**SEARCH STRATEGIES**			
Full search strategies	8	Include the search strategies for each database and information source, copied and pasted exactly as run.	P9-10, Supplement 1
Limits and restrictions	9	Specify that no limits were used, or describe any limits or restrictions applied to a search (e.g., date or time period, language, study design) and provide justification for their use.	P9
Search filters	10	Indicate whether published search filters were used (as originally designed or modified), and if so, cite the filter(s) used.	Supplement 1
Prior work	11	Indicate when search strategies from other literature reviews were adapted or reused for a substantive part or all of the search, citing the previous review(s).	P10

Updates	12	Report the methods used to update the search(es) (e.g., rerunning searches, email alerts).	P9
Dates of searches	13	For each search strategy, provide the date when the last search occurred.	P9
**PEER REVIEW**			
Peer review	14	Describe any search peer review process.	P10
**MANAGING RECORDS**			
Total Records	15	Document the total number of records identified from each database and other information sources.	P14, Figure 1
Deduplication	16	Describe the processes and any software used to deduplicate records from multiple database searches and other information sources.	P10

PRISMA-S: An Extension to the PRISMA Statement for Reporting Literature Searches in Systematic Reviews
Rethlefsen ML, Kirtley S, Waffenschmidt S, Ayala AP, Moher D, Page MJ, Koffel JB, PRISMA-S Group.
Last updated February 27, 2020.

(1) First Bayesian NMA of CBT for IBS to compute ESS/RSS for every indirect contrast.

(2) Added 18 RCTs, modeled continuous global symptoms, QoL, pain; quantified info-sufficiency per pair.

(3) Ranks digital, self-help, phone CBT top for symptom relief; reveals most contrasts lack adequate ESS.

(4) Validates immediate use of remote CBT; calls for large, low-bias trials to close evidence gap.
